# A Bioactive Oleogel Incorporating Chamomile Hydroglyceric Extract: Physicochemical Characterization, Occlusive Properties, Controlled Release and Biocompatibility

**DOI:** 10.3390/gels12080704

**Published:** 2026-08-06

**Authors:** Mona Luciana Gălățanu, Ion Mircioiu, Carmen Elisabeta Manea, Carmen Marinela Mihăilescu, Mirela Antonela Mihăilă, Roxana Colette Sandulovici, Marinela Bostan, Florin Adrian Marin, Adina Boldeiu, Mihaela Savin, Vasilica Țucureanu, Marian Ion, Emilia Pănuș, Oana Brincoveanu, Mariana Panțuroiu

**Affiliations:** 1 Faculty of Pharmacy, Titu Maiorescu University, 040314 Bucharest, Romania; luciana.galatanu@prof.utm.ro (M.L.G.); ion.mircioiu@prof.utm.ro (I.M.); mirela.mihaila@prof.utm.ro (M.A.M.); roxana.sandulovici@prof.utm.ro (R.C.S.); mariana.panturoiu@prof.utm.ro (M.P.); 2 Horia Hulubei National Institute for R&D in Physics and Nuclear Engineering (IFIN-HH), Reactorului 30 St., 077125 Magurele, Romania; 3 National Institute for Research and Development in Microtehnologies (IMT), 077190 Bucharest, Romania; adina.boldeiu@imt.ro (A.B.); mihaela.savin@imt.ro (M.S.); vasilica.tucureanu@imt.ro (V.Ț.); marian.ion@imt.ro (M.I.); oana.brincoveanu@imt.ro (O.B.); 4 Doctoral School, University of Medicine and Pharmacy of Craiova, 200349 Craiova, Romania; 5 Stefan S. Nicolau Institute of Virology, Mihai Bravu St, 030304 Bucharest, Romania; marinela.bostan@virology.ro; 6 Eminvest Pharmaceuticals SRL, Stefan Stoica St., 012243 Bucharest, Romania; florin.marin@eminvest.ro; 7 Department of Biochemistry, Faculty of Medicine, Ovidius University of Constanta, Universitatii Street No. 1, 900470 Constanta, Romania; temilia2@yahoo.com

**Keywords:** oleogel, *Matricaria chamomilla*, rheology, structural characterization, occlusive properties, controlled polyphenol release, biocompatibility

## Abstract

Oleogels have emerged as promising anhydrous semisolid systems for dermato-cosmetic and pharmaceutical applications owing to their improved physicochemical stability, enhanced occlusive properties, reduced dependence on preservatives, and ability to incorporate and deliver both lipophilic and dispersed hydrophilic bioactive compounds. The present study aimed to develop and comprehensively characterize a multifunctional lipid-based oleogel incorporating a *Matricaria chamomilla* hydroglyceric extract together with sea buckthorn oil and sweet almond oil as natural sources of antioxidant and skin-protective phytoconstituents for potential topical applications. The formulation was prepared by the melt method and comprehensively characterized for its physicochemical, functional, and biological properties. The chamomile extract exhibited a high phenolic content and pronounced antioxidant activity, while HPLC-DAD analysis confirmed the presence of characteristic phenolic acids and flavonoids. The developed oleogel showed good physicochemical stability under accelerated storage conditions, appropriate pH for topical application, favorable spreadability, pseudoplastic flow behavior with a Casson yield stress of 0.19 Pa, and a homogeneous microstructure with a mean droplet size of 5.4 ± 1.1 μm. The formulation also demonstrated pronounced occlusive properties (67.9 ± 0.3% and 56.4 ± 0.2% after 24 and 48 h, respectively), preserved the antioxidant activity of the incorporated phytoconstituents, and provided controlled biphasic release of phenolic compounds, reaching 64.9 ± 5.9% after 24 h in Franz diffusion studies. Furthermore, no cytotoxic effects were observed on HaCaT keratinocytes, confirming the good *in vitro* biocompatibility of the formulation. Overall, the developed oleogel successfully combined physicochemical stability, preserved antioxidant functionality, controlled phenolic release, pronounced occlusive properties, and biocompatibility, highlighting the potential of anhydrous oleogel systems as multifunctional topical delivery platforms for plant-derived bioactive compounds.

## 1. Introduction

In recent years, increasing attention has been directed toward the development of anhydrous semi-solid systems as alternatives to conventional emulsions in dermato-cosmetic and pharmaceutical formulations [1,2,3]. Among these systems, oleogels have emerged as promising platforms due to their ability to structure liquid oils into viscoelastic, three-dimensional networks using low concentrations of organogelators. This approach enables the design of lipid-based semi-solid formulations with improved physicochemical stability and a reduced need for preservatives, addressing current consumer and regulatory demands for safer and more sustainable topical products.

Oleogels are typically formed by the self-assembly or crystallization of low-molecular-weight gelators, such as waxes, fatty alcohols, or polymers, within a continuous oil phase. The resulting network immobilizes the liquid oil, leading to gel-like behavior characterized by dominant elastic properties. The type and concentration of organogelator, as well as the composition of the oil phase, play a critical role in determining the microstructure, rheological behavior, and functional performance of the system [4].

Recent studies have highlighted the potential of oleogels as delivery systems for lipophilic active compounds, including antioxidants, vitamins and anti-inflammatory agents [5,6,7]. Compared to traditional emulsions, oleogels offer enhanced oxidative stability, improved skin feel, and the ability to modulate the release profile of incorporated actives. Furthermore, the use of natural structuring agents, such as plant-derived waxes, aligns with the growing interest in green and clean-label formulations. Although oleogels are traditionally regarded as anhydrous systems, recent studies have demonstrated that limited amounts of dispersed polar phases can be successfully incorporated without altering the oleaginous nature of the continuous phase, provided that the structural integrity of the system is maintained by the organogel network [8]. This approach broadens the applicability of oleogels by enabling the incorporation of both lipophilic and hydrophilic bioactive compounds within a structured lipid matrix.

In this context, the selection of suitable bioactive oils and plant-derived extracts is of particular importance for the development of functional oleogel systems.

Sea buckthorn oil, a bioactive ingredient rich in carotenoids, phytosterols, and fatty acids, has attracted considerable interest due to its antioxidant, regenerative, and anti-inflammatory properties [9,10,11]. However, its incorporation into stable topical systems remains challenging due to its susceptibility to oxidation. Embedding such sensitive oils into an oleogel matrix may improve their stability and bioavailability while maintaining desirable sensory properties.

In addition, sweet almond oil is a widely used natural ingredient in dermato-cosmetic formulations due to its rich composition of unsaturated fatty acids, particularly oleic and linoleic acids, as well as tocopherols and phytosterols. These compounds contribute to its emollient, antioxidant, anti-inflammatory, and photoprotective properties [12,13,14]. In oleogel systems, almond oil acts as an effective carrier for lipophilic bioactives while improving the stability, spreadability, and sensory characteristics of the formulation.

Furthermore, chamomile (*Matricaria chamomilla* L.) extract is widely recognized for its rich phytochemical profile and beneficial effects on skin health. It contains bioactive compounds such as flavonoids, phenolic acids, and terpenoids, which contribute to its well-studied antioxidant, anti-inflammatory, antimicrobial, and soothing properties [15,16]. The use of a hydroglycerinic extract allows for the efficient extraction of both hydrophilic and moderately lipophilic compounds, maximizing the recovery of active constituents [17]. Moreover, chamomile extracts have been shown to enhance skin barrier function and may improve the transdermal penetration of active substances [18]. In oleogel-based systems, the incorporation of such extracts can further enhance functional performance by combining lipid-based delivery with additional bioactivity from the polar phase.

To obtain a stable oleogel system capable of incorporating both lipophilic and polar bioactive compounds, appropriate structuring and emulsifying agents were selected to ensure network formation and formulation stability. Additionally, Caprylic/Capric Triglyceride (and) Hydrogenated Rapeseed Oil (Softisan^®^ Pura) was included as an emollient and structuring agent to enhance the texture and stability of the system. Polysorbate 80 was used as a co-emulsifier to facilitate the incorporation of the polar phase, while white wax acted as the primary organogelator, contributing to the formation of the three-dimensional network structure of the oleogel. The combination of the selected lipid phase and the beeswax network was also expected to modulate the diffusion of the incorporated phytoconstituents, thereby contributing to sustained topical release.

Despite the increasing number of studies on oleogels [19,20,21], research integrating formulation development, physicochemical characterization and biological evaluation using cell-based model remains limited.

In this context, the present study aims to develop and comprehensively characterize a biocompatible oleogel based on a combination of natural emollients, namely sea buckthorn and sweet almond oil, structured with white wax and enriched with chamomile hydroglyceric extract. The rationale behind this formulation was to combine the antioxidant and skin-protective properties of plant-derived phytoconstituents with the intrinsic barrier-forming capacity of oleogels to obtain a multifunctional topical delivery system.

Maintaining a moist microenvironment is recognized as a key factor in effective wound healing, as it promotes cell migration, tissue regeneration, and re-epithelialization while reducing transepidermal water loss. Owing to their three-dimensional lipid network, oleogels represent attractive topical platforms capable of simultaneously forming a protective barrier, maintaining skin hydration, and enabling the sustained delivery of bioactive compounds.

Although chamomile extracts and oleogel-based formulations have been individually investigated, studies combining hydroglyceric chamomile extracts with bioactive vegetable oils in a beeswax-structured oleogel remain scarce. To the best of our knowledge, no previous study has comprehensively investigated a biphasic oleogel combining a hydroglyceric chamomile extract with sea buckthorn and sweet almond oils while simultaneously evaluating its structural, physicochemical, functional, release, and biological properties. The present approach was designed to integrate the antioxidant properties of chamomile with the barrier-supporting and emollient effects of sea buckthorn and sweet almond oils in an anhydrous lipid matrix. Compared with conventional oil-in-water creams or emulgels, anhydrous oleogel systems offer improved physicochemical stability, enhanced occlusive properties, reduced dependence on preservatives, and the potential for sustained release of plant-derived bioactive compounds.

To validate this formulation concept, the developed oleogel was comprehensively evaluated using complementary physicochemical, structural, functional, and biological approaches. The oleogel was characterized in terms of phytochemical composition, rheological behavior, spreadability, pH, accelerated stability, occlusive performance, and antioxidant activity. In addition, dynamic light scattering, zeta potential analysis, Fourier transform infrared spectroscopy, and scanning electron microscopy were employed to investigate the structural organization, colloidal stability, and chemical integrity of the lipid matrix. The release behavior of phenolic compounds was assessed using a Franz diffusion cell, while *in vitro* biocompatibility was evaluated on HaCaT keratinocytes.

By integrating structural characterization with functional and biological evaluation, this study provides a comprehensive assessment of the potential of a chamomile-based oleogel as a multifunctional topical delivery platform with potential applications in dermato-cosmetic formulations, skin barrier restoration, and wound care.

## 2. Results and Discussion

### 2.1. Characterization of the Natural Oils Used as the Oleogel Base

#### 2.1.1. Physicochemical Quality Assessment of Sea Buckthorn and Sweet Almond Oils

Prior to oleogel preparation, the physicochemical quality of sea buckthorn oil and sweet almond oil was evaluated to verify their suitability as lipid components of the formulation. The assessment included refractive index, relative density, acid value, saponification value, iodine value, and peroxide value, which are widely accepted indicators of oil identity, purity, and oxidative stability. The results are summarized in Table 1, expressed as mean ± standard deviation for statistical comparison.

As shown in Table 1, all measured parameters were within the reference ranges reported for sea buckthorn and sweet almond oils, confirming their satisfactory physicochemical quality. The relatively low peroxide values indicate that both oils had undergone minimal oxidative degradation prior to formulation, while the acid values suggest a limited content of free fatty acids, consistent with good-quality vegetable oils. The refractive index, relative density, iodine value, and saponification value were also in agreement with the expected characteristics of the selected oils, supporting their suitability for incorporation into the oleogel formulation.

#### 2.1.2. Fatty Acid Profile of Sea Buckthorn and Sweet Almond Oils by GC–MS

The GC–MS profiles of sea buckthorn oil and sweet almond oil are summarized in Table 2 while representative chromatograms are shown in Figures S1 and S2 (Supplementary Materials). Sea buckthorn oil was mainly characterized by a high content of palmitoleic and palmitic acids, whereas sweet almond oil was dominated by oleic and linoleic acids. These fatty acid profiles are consistent with literature data and support the complementary functional roles of both oils in the oleogel formulation, contributing to skin barrier support, emollient properties, and oxidative stability.

The markedly higher content of palmitoleic acid in sea buckthorn oil represents a distinctive feature of this lipid source and may contribute to its recognized skin-regenerating and barrier-supporting properties. Meanwhile, the predominance of oleic and linoleic acids in sweet almond oil is associated with emollient activity and improved skin hydration, making both oils suitable components for topical oleogel formulations.

### 2.2. Phytochemical Characterization of the Chamomile Hydroglyceric Extract

The chamomile hydroglyceric extract prepared in the laboratory was phytochemically characterized prior to its incorporation into the oleogel formulation in order to identify its major bioactive constituents and evaluate its antioxidant potential. Comprehensive characterization included qualitative and quantitative HPLC-DAD-MS/MS analysis, determination of the total polyphenol content, and assessment of antioxidant activity using the ABTS radical scavenging assay. These analyses provided the phytochemical basis for interpreting the biological performance of the developed oleogel.

#### 2.2.1. HPLC-DAD Analysis of the Chamomile Hydroglyceric Extract

HPLC analysis confirmed the presence of several phenolic acids and flavonoids in the chamomile hydroglyceric extract (Table 3; Figure S3, Supplementary Materials). The predominant constituents included p-coumaric acid, apigenin-7-O-glucoside, and rutin, together with other minor phenolic compounds. This phytochemical profile is consistent with previous reports for chamomile extracts and supports the antioxidant and skin-protective potential of the developed oleogel.

#### 2.2.2. Total Polyphenol Content

The total phenolic content (TPC) of the chamomile hydroglyceric extract was determined using the Folin–Ciocalteu assay and expressed as gallic acid equivalents (GAE). The extract contained 0.690 ± 0.001 mg GAE/mL, corresponding to 6.90 ± 0.01 mg GAE/g of dried chamomile flowers.

The obtained TPC is comparable with values reported for aqueous and glycerol-containing chamomile extracts, although it is generally lower than those reported for concentrated hydroethanolic or methanolic extracts [22,23]. This difference is expected and primarily reflects the influence of the extraction solvent, extraction conditions, and the expression of results. Hydroethanolic solvents generally provide higher extraction efficiencies for phenolic compounds, whereas glycerol–water mixtures are selected to obtain biocompatible extracts suitable for direct incorporation into topical formulations without the need for solvent removal [24].

Although the hydroglyceric extraction system may yield a lower total phenolic content than hydroethanolic extraction, it offers important technological and dermatological advantages, including low toxicity, high skin compatibility, and excellent moisturizing properties. Consequently, the measured phenolic content represents a satisfactory compromise between extraction efficiency and formulation compatibility, while remaining sufficient to provide antioxidant activity and to support the biological performance of the developed oleogel.

The HPLC-DAD analysis and total phenolic content determination provided an appropriate phytochemical characterization of the chamomile hydroglyceric extract for formulation development. Quantitative chromatographic standardization using selected phytochemical markers represents an important avenue for future research, contributing to enhanced extract characterization, quality control, and batch-to-batch reproducibility.

#### 2.2.3. Antioxidant Activity of Chamomile Hydroglyceric Extract

**Figure 1:**
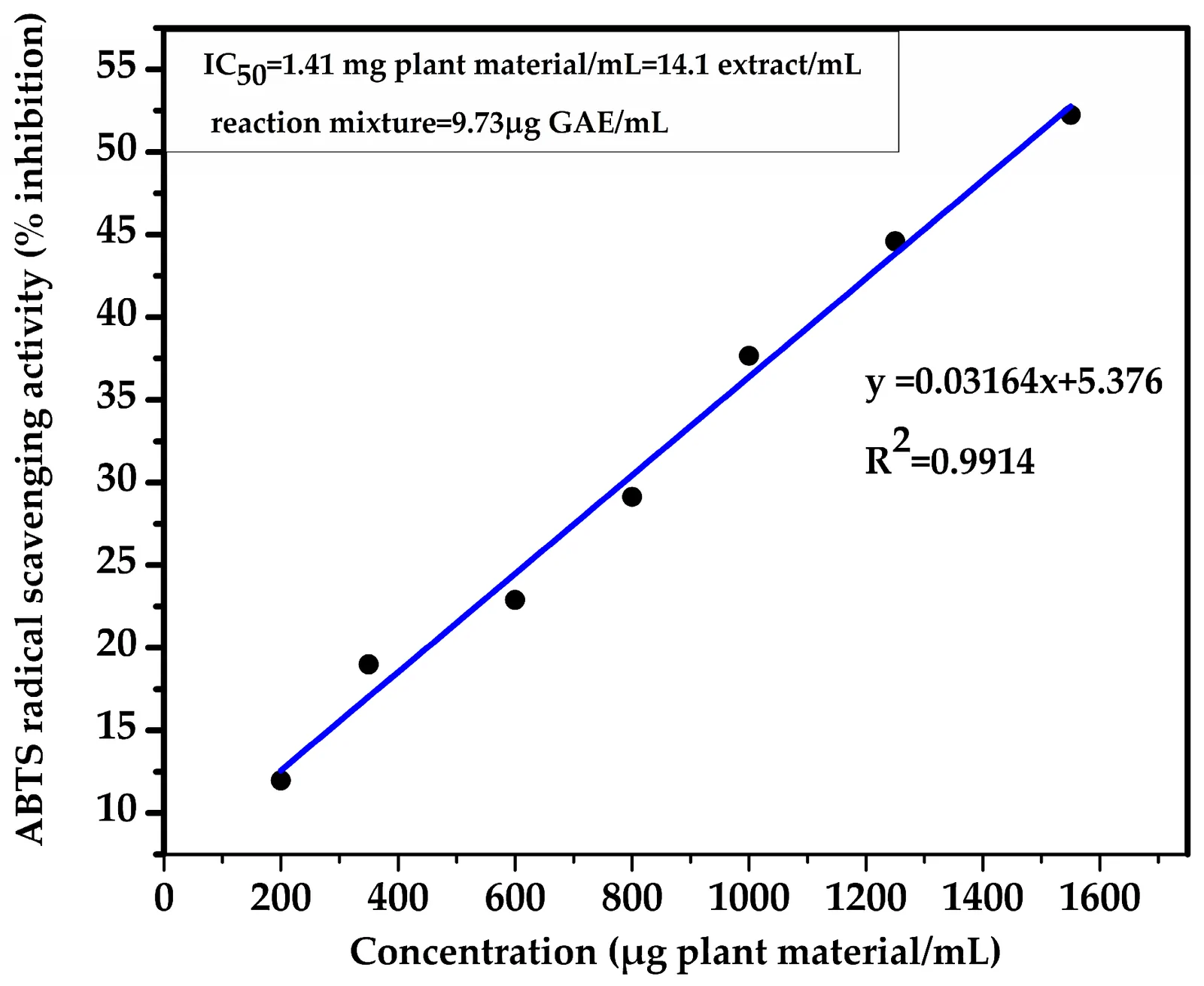
Concentration-dependent antioxidant activity of the chamomile hydroglyceric extract determined by the ABTS assay.

The antioxidant activity of the chamomile hydroglyceric extract was evaluated using the ABTS radical scavenging assay. A concentration-dependent increase in radical scavenging activity was observed over the investigated concentration range, with inhibition values increasing from 11.98 ± 0.01% at 200 μg plant material/mL to 52.24 ± 0.03% at 1509 μg plant material/mL (Figure 1). Linear regression analysis showed an excellent correlation between extract concentration and ABTS radical scavenging activity (R^2^ = 0.9914), and the IC_50_ value was calculated as 1.41 ± 0.01 mg plant material/mL, corresponding to 14.1 ± 0.01 μL hydroglyceric extract/mL of reaction mixture or approximately 9.73 ± 0.01 μg GAE/mL.

The antioxidant activity observed is in agreement with both the HPLC-DAD profile and the total phenolic content of the extract. Phenolic acids and flavonoids identified in the extract, particularly p-coumaric acid, chlorogenic acid, rutin hydrate, quercetin, and apigenin-7-O-glucoside, are well known for their ability to donate hydrogen atoms or electrons and to efficiently neutralize free radicals [22,25]. Their combined action is therefore considered the main contributor to the ABTS radical scavenging activity of the extract.

The obtained IC_50_ value is comparable with those reported for glycerol-containing chamomile extracts, while remaining higher than the values generally described for concentrated hydroethanolic or methanolic extracts. Such differences are expected because the extraction efficiency of glycerol–water mixtures towards phenolic compounds is lower than that of hydroalcoholic solvents. However, hydroglyceric extracts offer important advantages for topical formulations, including excellent skin compatibility, intrinsic moisturizing properties, and the absence of organic solvent residues, allowing their direct incorporation into dermatological and cosmetic formulations without additional solvent removal or purification steps.

Overall, the combination of a characteristic phenolic profile, a measurable total phenolic content, and a moderate but concentration-dependent antioxidant activity demonstrates that the chamomile hydroglyceric extract represents a suitable bioactive ingredient for the developed oleogel. The antioxidant properties of the extract are expected to contribute to the protection of the skin against oxidative stress, complementing the emollient and barrier-restoring effects provided by the lipid phase of the formulation.

### 2.3. Oleogel Characterization

#### 2.3.1. Organoleptic Evaluation

The organoleptic characteristics of the developed oleogel, as evalaute by five trained evaluators, are summarized in Table 4. The formulation exhibited a homogeneous semi-solid appearance, characteristic yellow-orange color, good consistency, and excellent spreadability throughout storage. Overall, the results indicate good sensory acceptability and preservation of the physical integrity of the formulation. Detailed organoleptic observations together with the corresponding radar plot are presented in Figure S5 (Supplementary Materials).

The radar chart (Figure S5, Supplementary Materials) further illustrates the balanced sensory profile of the oleogel and supports its suitability for topical application.

#### 2.3.2. pH Determination

**Figure 2:**
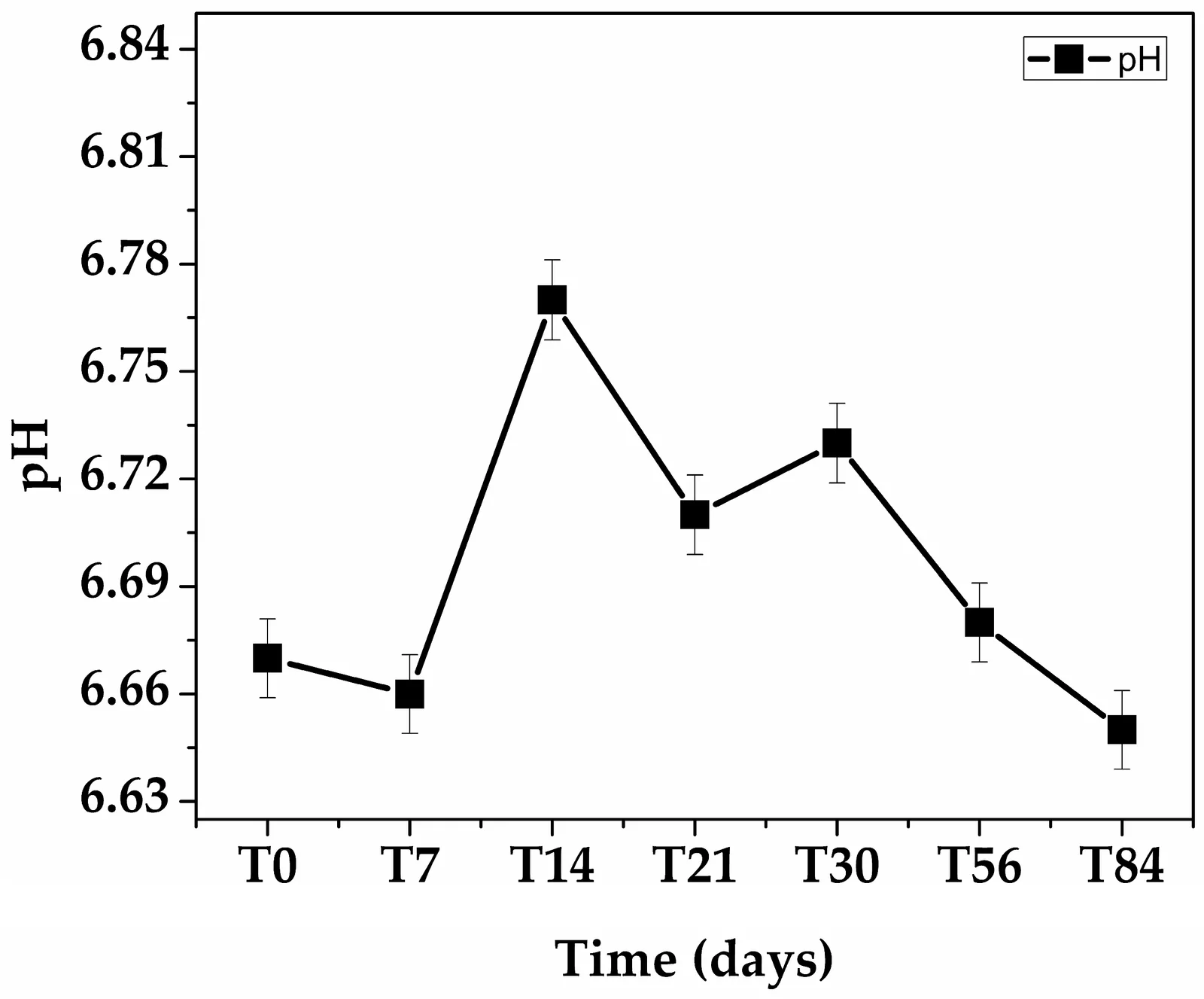
pH stability of the developed oleogel during the 84 days storage period. Values are expressed as mean ± SD (*n* = 3).

The pH of the oleogel remained stable throughout the 84-day storage period (Figure 2, Table S1), with only minor fluctuations that were within the expected experimental variability. The obtained values remained within the physiological range suitable for topical application, indicating good physicochemical stability of the formulation during storage.

#### 2.3.3. Physical Stability

The physical stability of the oleogel was evaluated by complementary accelerated stability tests, including centrifugation, storage under different temperature conditions (4 ± 2 °C, 25 ± 2 °C and 37 ± 2 °C), and five freeze–thaw cycles. Throughout all evaluations, the formulation maintained its structural integrity without visible phase separation, oil leakage, or irreversible changes in appearance. Detailed results for each stability test are presented in Tables S2–S4 (Supplementary Materials).

A slight increase in firmness was observed after storage at low temperature, whereas a minor decrease in consistency occurred under accelerated thermal conditions. However, these changes were reversible and did not affect the overall homogeneity or stability of the formulation. Likewise, repeated freeze–thaw cycles produced no evidence of structural breakdown or oil exudation, indicating that the crystalline network remained stable under mechanical and thermal stress.

The observed stability is consistent with previous reports on topical oleogel systems, in which appropriately structured lipid networks successfully preserved their integrity under mechanical and thermal stress. Alongi et al. reported that olive oil oleogels structured with waxes or phytosterols maintained their structural properties during prolonged storage, whereas ethyl cellulose-based systems showed greater susceptibility to structural changes [26]. Similarly, Sheta et al. demonstrated that dibenzalacetone-loaded sunscreen oleogels remained physically stable after centrifugation, cooling–heating, and freeze–thaw cycles [27], while Łętocha et al. reported that cosmetic oleogels containing probiotic-loaded alginate microspheres successfully withstood centrifugation and thermal stability testing without phase separation [28]. Although direct comparisons should be interpreted with caution because of differences in oil composition, gelator type, active ingredients, and experimental protocols, the comparable stability observed in the present study suggests that the beeswax-based crystalline network effectively immobilized the liquid oil phase and preserved the structural integrity of the formulation under accelerated storage conditions.

Overall, these characteristics demonstrates the suitability of the developed oleogel for storage, transportation, and potential topical applications. The excellent physical stability observed in the present study further supports one of the principal advantages of the anhydrous oleogel system over conventional emulsion-based topical formulations, namely the ability to maintain structural integrity without the instability commonly associated with aqueous continuous phases.

#### 2.3.4. Droplet Size

**Figure 3:**
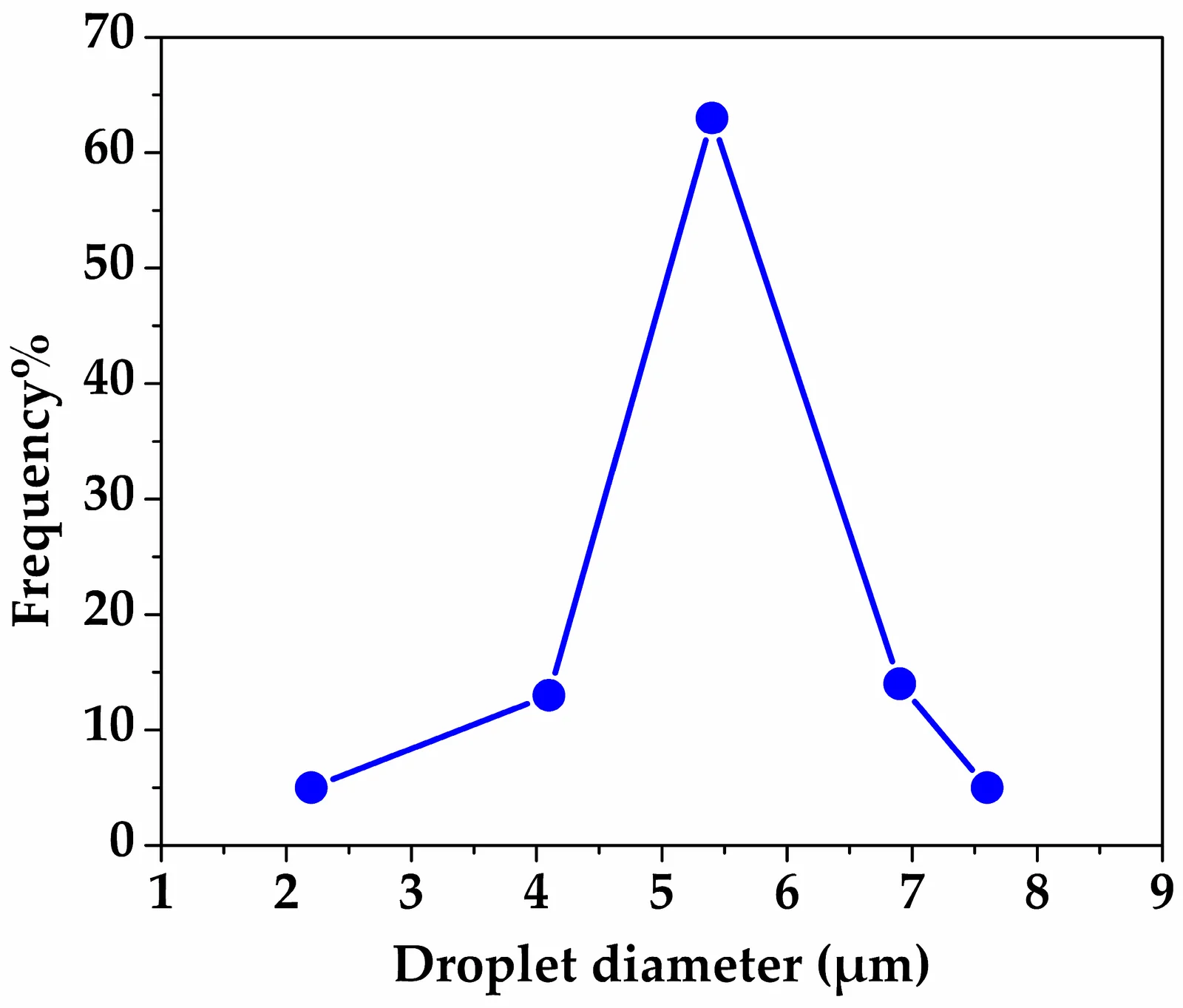
Frequency distribution of droplet diameters in the developed oleogel system based on the measurement of 100 individual droplets (n = 100), showing a unimodal distribution centered around the mean droplet diameter (5.39 ± 1.13 µm).

Figure 3 presents the droplet size distribution along with representative micrographs of the developed system. The analysis revealed a mean droplet diameter of 5.39 ± 1.13 µm, and a polydispersity index (PDI) of 0.044, indicating a relatively narrow and homogeneous size distribution of the dispersed polar droplets within the oleogel matrix. These values reflect the size of the internal hydroglyceric phase droplets stabilized within the lipid continuous phase. The measurements were performed on a total of 100 droplets (n = 100), ensuring the statistical relevance of the obtained data.

Similar droplet sizes have been reported in oleogel-based systems stabilized with triterpenoid saponins, where mean diameters ranged between 4.89 and 6.82 µm [29], or in a sunflower wax-based oleogel emulsion, where droplet sizes of approximately 4.7 was observed [30]. In contrast, other studies reported smaller droplet sizes (e.g., <3 µm), particularly when high-energy emulsification methods were employed [31,32].

The low PDI value (0.044) indicates a narrow droplet size distribution and is consistent with literature data for stable emulsion-based oleogel systems, which typically exhibit low polydispersity [33,34].

#### 2.3.5. Rheological Behavior

##### Viscosity and Flow Behavior

**Figure 4:**
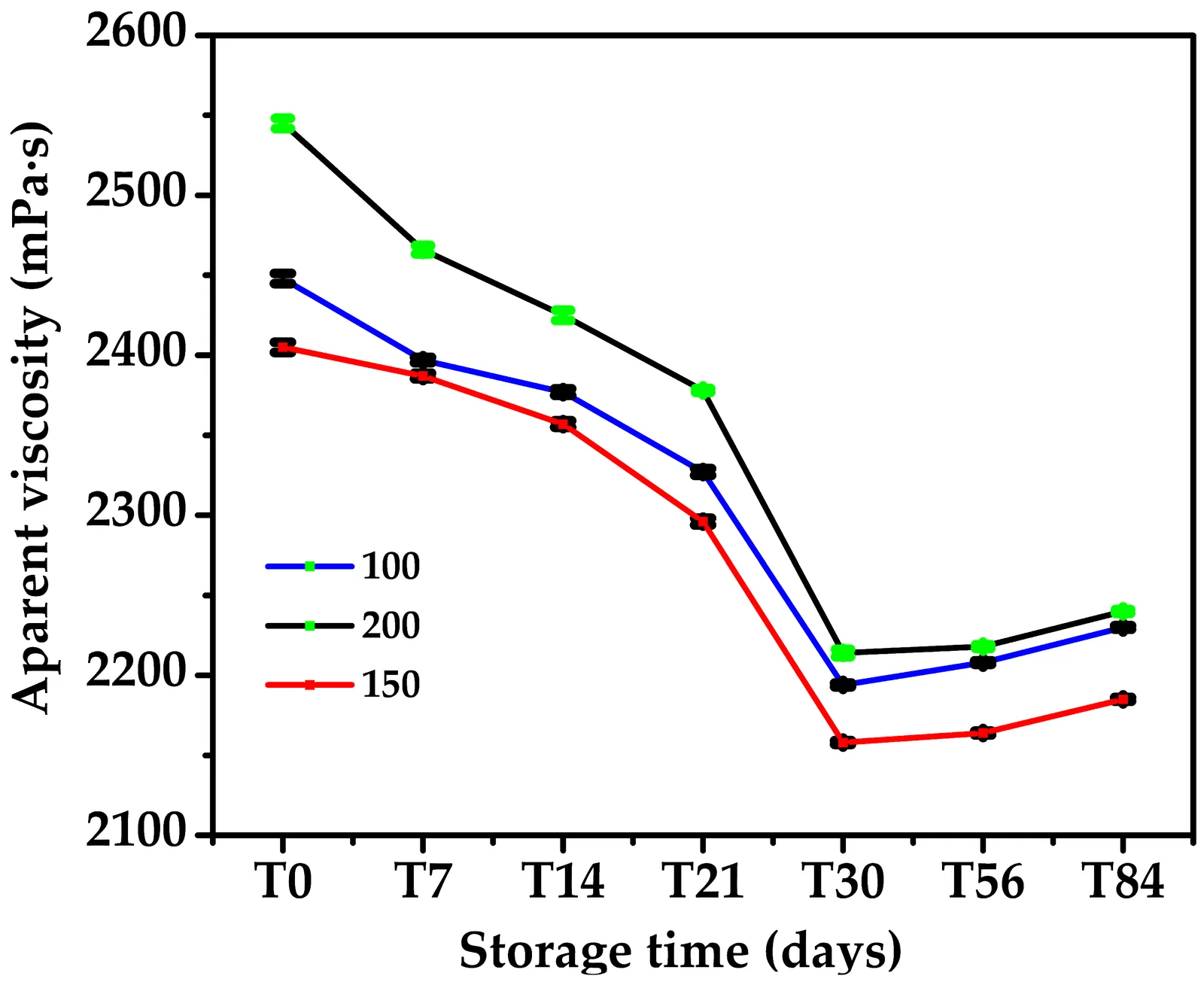
Viscosity evolution of the oleogel over time at different rotational speeds (100, 150 and 200 rpm). Values are expressed as mean ± SEM (n = 3). Error bars represent the standard error of the mean (SEM); where not clearly visible, they are smaller than the corresponding data point symbols.

The viscosity of the oleogel was monitored during 84 days of storage at three rotational speeds (100, 150 and 200 rpm) (Figure 4; Table S4). A moderate decrease in apparent viscosity was observed during storage, followed by a slight increase after day 56, suggesting progressive reorganization of the crystalline network. Despite these minor variations, the formulation maintained relatively high viscosity values throughout the study, indicating preservation of its structural integrity.

The decrease in viscosity with increasing rotational speed confirms the non-Newtonian shear-thinning behavior of the oleogel, which is desirable for topical formulations because it combines good stability during storage with easy spreading during application.

##### Yield Stress

**Figure 5:**
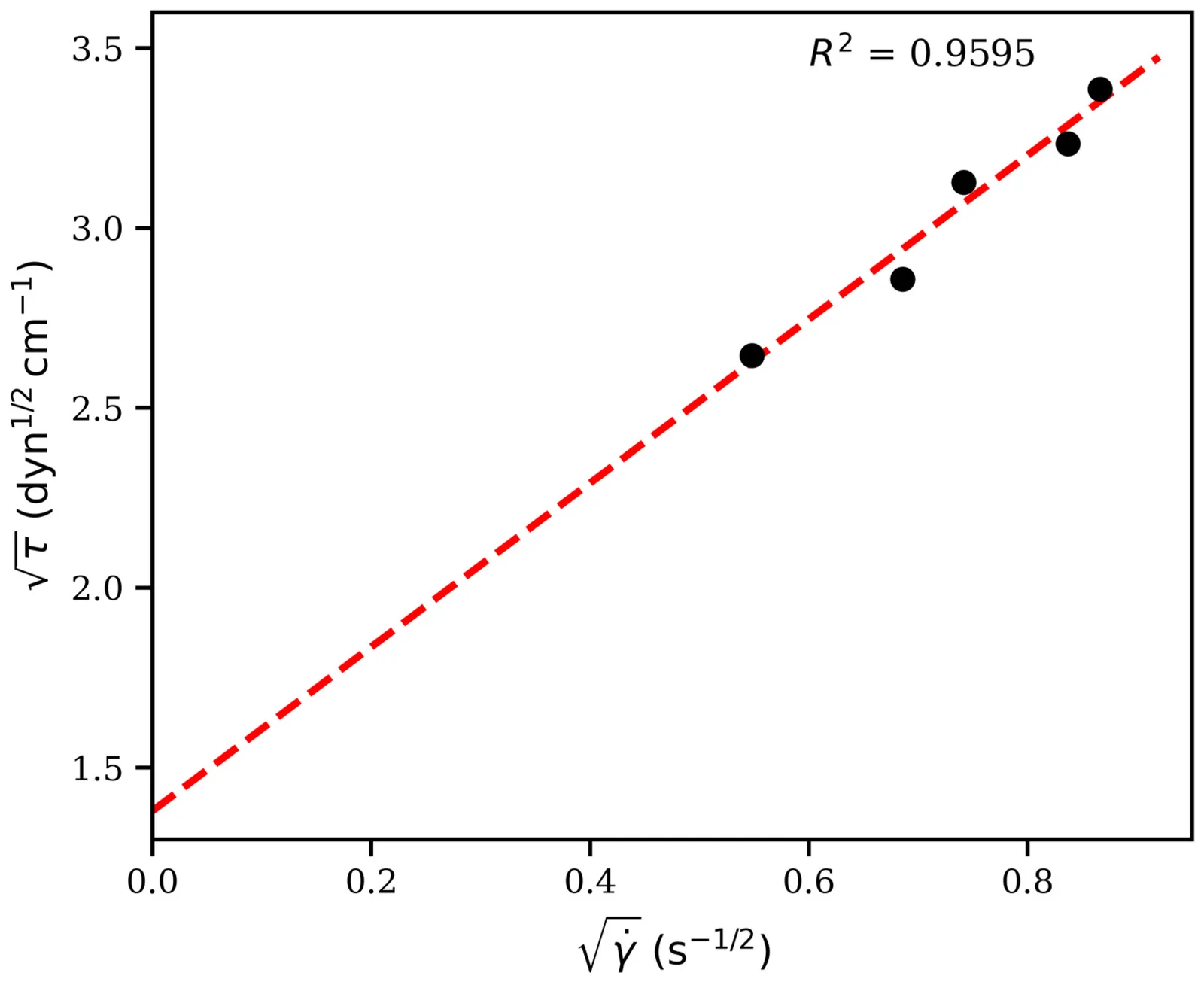
Linearized Casson plot obtained from rheological measurements of the developed oleogel. The linear regression showed a good fit (R^2^ = 0.9595) and yielded a Casson yield stress (τ_0_) of 0.1905 Pa, confirming the yield-stress behavior of the formulation.

To further characterize the mechanical behavior of the oleogel and evaluate the strength of its internal network, the experimental rheological data were analyzed using the Casson model. The relationship between shear stress and shear rate is presented in Figure S6 (Supplementary Materials), which shows a non-linear profile characteristic of pseudoplastic materials. As the applied shear rate increased, the shear stress increased in a non-proportional manner, indicating that the oleogel does not behave as a Newtonian fluid but rather as a structured semisolid system. This behavior is typical of oleogels, where a three-dimensional crystalline network immobilizes the liquid oil phase and gradually breaks down under applied stress [35]. For quantitative evaluation, the experimental data were transformed according to the linearized Casson equation and fitted by linear regression. The resulting Casson plot is shown in Figure 5, while the experimental data used for model fitting are provided in Table S5 (Supplementary Materials). The linear fit exhibited a good coefficient of determination (R^2^ = 0.9595), supporting the suitability of the Casson model for describing the rheological behavior of the developed oleogel. Details of the Casson model calculations, including the determination of the yield stress and plastic viscosity from the linear regression parameters, are presented in the Supplementary Materials.

The calculated Casson yield stress (τ_0_) was 0.1905 Pa, whereas the Casson plastic viscosity (ηc) was 0.5189 Pa·s. These values indicate the presence of a well-developed crystalline network capable of resisting deformation under low applied stresses. The yield stress represents the minimum stress required to initiate flow and reflects the strength of the interactions between the crystalline domains and the immobilized oil phase. Below this critical stress, the oleogel behaves as a structured semisolid, whereas above τ_0_ the network progressively breaks down, allowing the material to flow and spread easily during topical application.

The observed yield-stress behavior is particularly advantageous for topical formulations, as it combines physical stability during storage with adequate spreadability during application. Furthermore, the pseudoplastic nature of the oleogel, evidenced by the decrease in apparent viscosity with increasing shear rate, contributes to improved sensory properties and user acceptability. Similar rheological characteristics have been reported for wax-structured oleogels and lipid-based semisolid systems intended for dermal administration, where the presence of a measurable yield stress is considered a key indicator of formulation robustness and structural stability [36].

##### Structural Recovery

The three-interval thixotropy test revealed a marked decrease in viscosity following the application of high shear stress (Tables S6 and S7 from Supplementary Materials). The viscosity decreased from 2235.67 mPa·s during the initial stage to 1341.33 mPa·s after the breakdown interval, corresponding to a viscosity loss of 40.01%. This reduction confirms the temporary disruption of the oleogel network under mechanical stress.

Following shear cessation, the viscosity progressively increased throughout the recovery stage, reaching 2611.33 mPa·s after 10 min. The calculated structural recovery was 116.80%, indicating complete restoration of the initial viscosity and a slight post-shear strengthening of the network. Similarly, the recovery efficiency reached 141.96%, suggesting that the oleogel structure not only recovered but also underwent further reorganization after stress removal.

This behavior may be attributed to the re-establishment of intermolecular interactions and the rearrangement of crystalline domains within the oleogel matrix. Similar observations have been reported for structured lipid systems, where the refinement and densification of the crystal network contribute to enhanced thixotropic recovery and restoration of mechanical properties after shear-induced disruption [37,38]. Such rapid and efficient structural recovery is particularly advantageous for topical applications, as the formulation can spread easily during application while rapidly regaining its consistency after deposition on the skin.

#### 2.3.6. Spreadability

**Figure 6:**
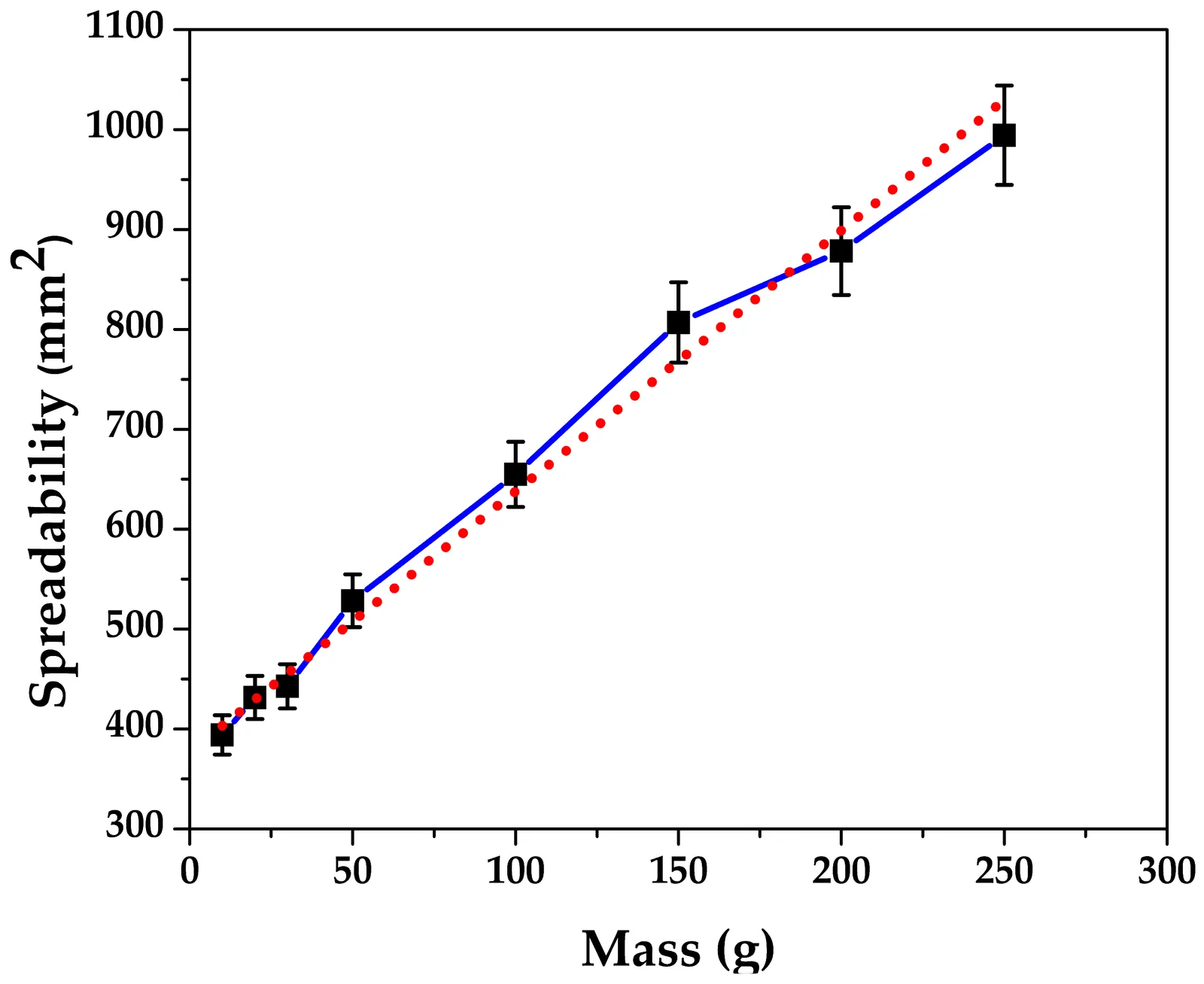
Spreadability of the oleogel as a function of the applied mass. Experimental data are presented as mean ± SE (n = 3). Where not clearly visible, error bars are smaller than the data point symbols. The solid blue line connects the experimental mean values (guide to the eye), whereas the red dotted line represents the linear regression fit (R^2^ = 0.99).

The spreadability of the oleogel increased progressively with increasing applied mass (Figure 6, Table S8 from Supplementary Materials), indicating a gradual deformation of the system under compression. The spreading area increased from 394.08 mm^2^ at 10 g to 983.15 mm^2^ at 250 g. The observed non-linear relationship suggests that the oleogel becomes progressively more deformable as the applied load increases, while maintaining structural coherence throughout the tested mass range.

Collectively, the results demonstrate favorable spreading properties of the oleogel, which may facilitate uniform application on the skin while preserving the structural integrity of the formulation.

#### 2.3.7. Structural Characterization

##### Dynamic Light Scattering (DLS) and Zeta Potential Analysis

**Figure 7:**
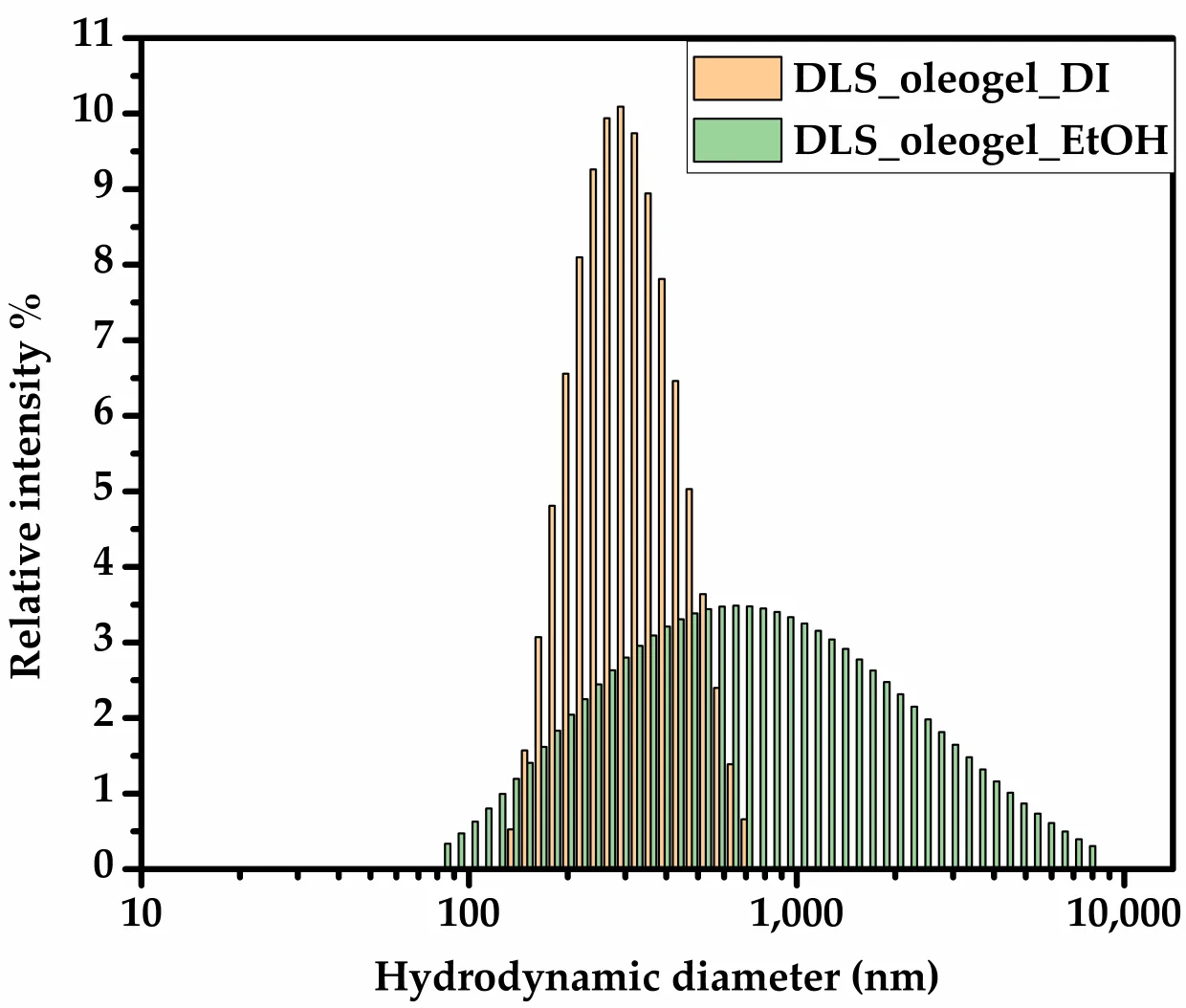
Hydrodynamic diameter distribution of the oleogel dispersed in distilled water (DI) and ethanol (EtOH), determined by dynamic light scattering (DLS). The aqueous dispersion exhibited a narrower and more homogeneous particle size distribution, while the ethanolic dispersion showed broader distributions shifted toward larger hydrodynamic diameters, suggesting solvent-dependent organization of the oleogel network.

Dynamic light scattering (DLS) and zeta potential analyses were performed in order to evaluate the colloidal behavior and dispersion stability of the oleogel system after dilution in two different media, namely distilled water (DI) and ethanol (EtOH). The obtained particle size distributions are presented in Figure 7 and reveal significant differences depending on the dispersion medium.

The oleogel dispersed in distilled water exhibited a narrower and more homogeneous size distribution, with the majority of the particles centered in the submicrometric range (approximately 250–500 nm). The relatively sharp unimodal distribution suggests the formation of more compact and uniformly dispersed aggregates in the aqueous environment. This behavior may be attributed to the partial organization of the amphiphilic components of the oleogel and the stabilization of smaller dispersed structures through hydrogen bonding and interfacial interactions.

In contrast, the oleogel dispersed in ethanol showed a broader distribution extending toward larger hydrodynamic diameters, reaching several micrometers. The broader profile indicates the presence of larger aggregates and a higher degree of polydispersity compared to the aqueous dispersion. This behavior can be associated with the different solvent polarity and the partial swelling or relaxation of the oleogel network in ethanol, which may promote the formation of larger dispersed domains.

The shift toward higher hydrodynamic diameters in ethanol suggests that the solvent influences the intermolecular interactions within the oleogel matrix, particularly those involving lipid components and structuring agents. Ethanol may partially disrupt weak intermolecular associations, leading to a more flexible network structure and consequently larger apparent particle sizes measured by DLS.

Despite these differences, both dispersions exhibited continuous particle size distributions without evidence of severe aggregation or macroscopic instability. Zeta potential measurements further supported the different colloidal behaviors observed in the two media. The aqueous dispersion displayed a negative ζ-potential of −34.33 mV, indicative of good electrostatic stabilization, whereas the ethanolic dispersion showed a near-neutral ζ-potential of +0.63 mV, suggesting that its stability is governed primarily by steric and intermolecular interactions rather than electrostatic repulsion. The high negative zeta potential may originate from ionizable fatty acids and phenolic constituents present within the oleogel formulation. The corresponding zeta potential distributions are presented in the Supplementary Materials (Figures S7 and S8).

Generally, absolute zeta potential values above approximately 30 mV are considered indicative of good colloidal stability. Therefore, the measured ζ-potential value in water suggests sufficient interparticle repulsion to maintain the physical stability of the dispersed oleogel system. The combined DLS and ζ-potential results indicate that the oleogel forms stable dispersed structures with solvent-dependent organization, supporting its suitability as a topical delivery system. The smaller and more homogeneous particles observed in the aqueous medium may additionally favor improved surface coverage and interaction with hydrated biological tissues, while the larger structures observed in ethanol may reflect increased swelling and solvent penetration within the oleogel network [39,40].

##### Fourier Transform Infrared Spectroscopy (FTIR)

**Figure 8:**
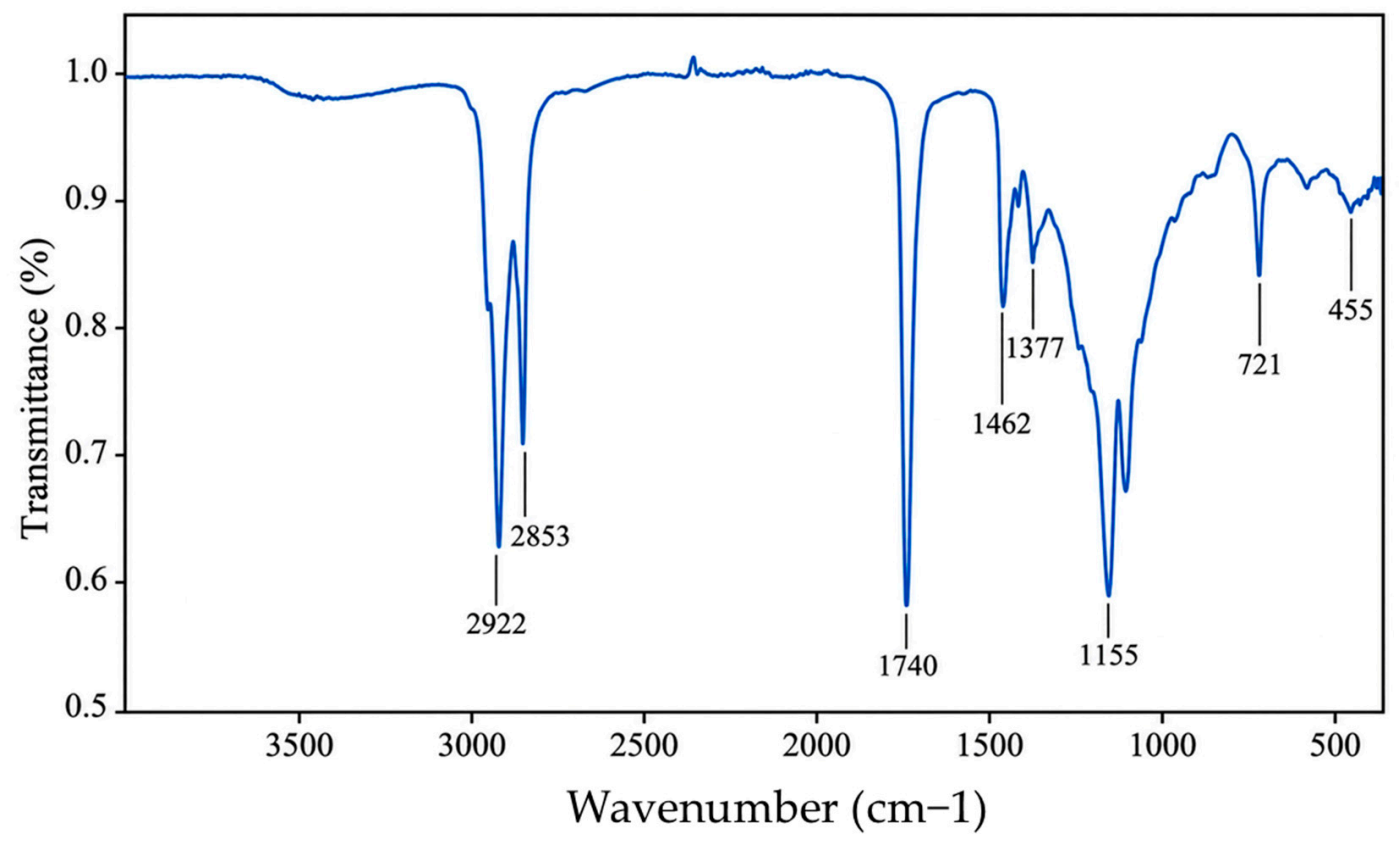
FTIR spectrum of the oleogel formulation recorded in ATR mode.

The FTIR spectra of both the bulk oleogel and the oleogel deposited on silicon are presented in Figure 8. The spectra exhibited the characteristic absorption bands of lipid-rich formulations, confirming the presence of the main components used in the oleogel preparation.

The intense absorption bands observed at approximately 2922 and 2853 cm^−1^ were assigned to the asymmetric and symmetric stretching vibrations of aliphatic CH_2_ groups, respectively. These bands are characteristic of long hydrocarbon chains present in sea buckthorn oil, sweet almond oil, caprylic/capric triglycerides, Bis-Diglyceryl Polyacyladipate-2, and white wax [41,42].

A strong absorption band centered at approximately 1740 cm^−1^ was attributed to the stretching vibration of ester carbonyl groups (C=O), confirming the presence of triglycerides and ester-based emollients within the formulation [34,43].

However, a contribution from chamomile-derived constituents containing carbonyl functionalities, such as matricine and apigenin derivatives, cannot be excluded, as similar absorption bands have been reported for *Chamomilla matricaria* extracts in the 1734–1712 cm^−1^ region [44].

Additional bands located at 1462 and 1377 cm^−1^ were associated with CH_2_ bending and CH_3_ deformation vibrations of lipid chains, while the absorption band observed around 1155 cm^−1^ corresponded to C–O–C stretching vibrations of ester functionalities [32,45]. The absorption band detected at approximately 721 cm^−1^ was assigned to CH_2_ rocking vibrations, which are characteristic of long-chain hydrocarbons and ordered lipid packing within semi-solid lipid systems [33].

The spectrum recorded for the oleogel deposited on silicon (Figure S9, Supplementary Materials) displayed additional absorption features in the region between 950 and 850 cm^−1^, which may be attributed to Si–O-related vibrations originating from the substrate.

In addition to the characteristic lipid-associated bands, a broad absorption band centered at approximately 3361 cm^−1^ was observed (Figure S8, Supplementary Materials), which may be attributed to O–H stretching vibrations of phenolic compounds and hydrogen-bonded hydroxyl groups originating from the *Chamomilla matricaria* extract. Furthermore, the band detected at approximately 1606 cm^−1^ may be associated with aromatic C=C vibrations of flavonoid and polyphenolic constituents present in the plant-derived extract. Similar bands have been reported for chamomile extracts rich in polyphenolic constituents. Nevertheless, no significant shifts were observed for the principal lipid-associated bands, indicating that deposition onto silicon did not alter the chemical structure of the oleogel components. The preservation of the characteristic ester and hydrocarbon bands demonstrates that the formulation maintained its chemical integrity after deposition on the substrate. Overall, the FTIR results confirm the successful incorporation of the lipidic components within the oleogel matrix and indicate the absence of detectable chemical degradation or undesired interactions between the formulation constituents and the silicon support.

##### Scanning Electron Microscopy (SEM)

**Figure 9:**
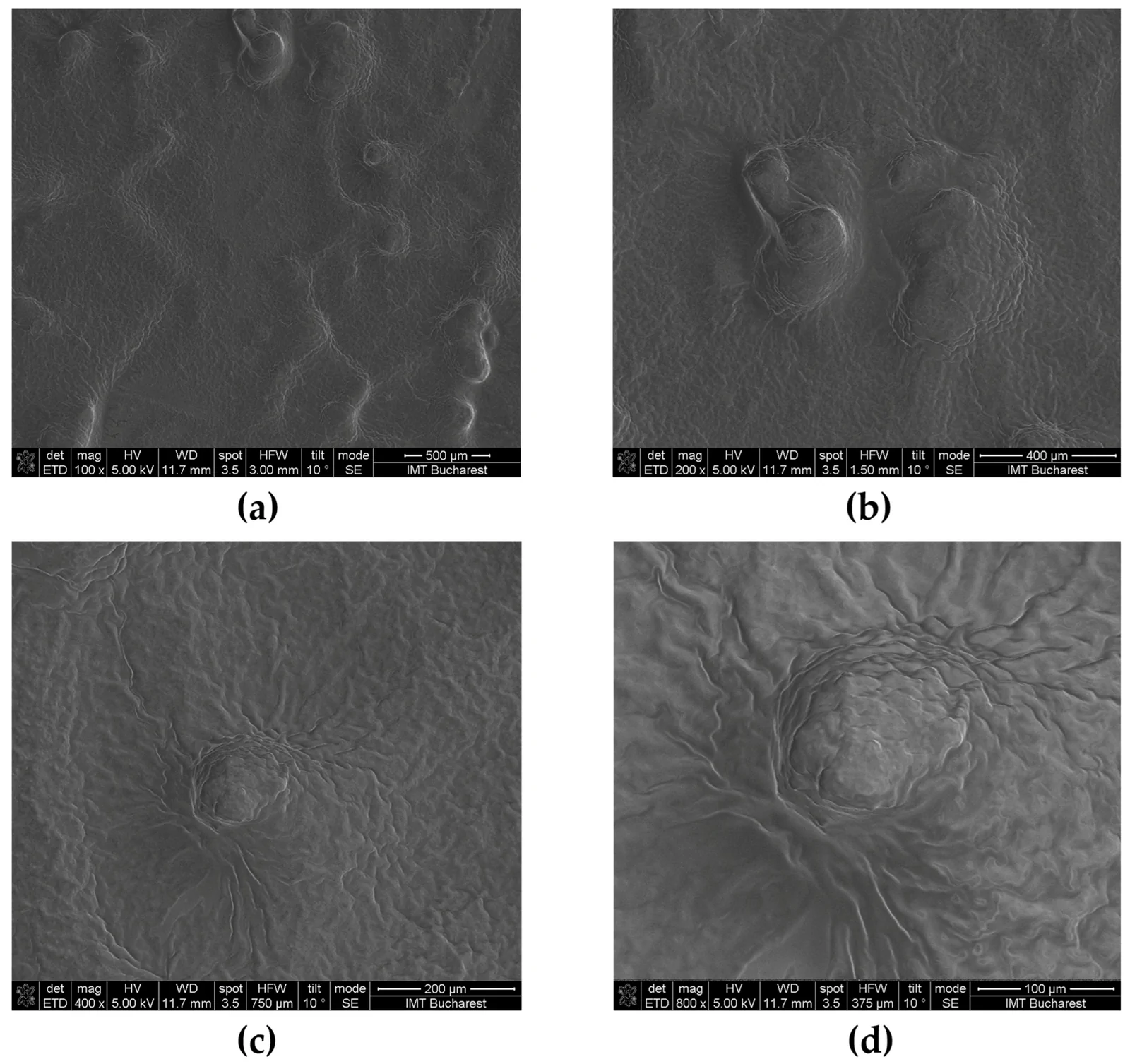
SEM micrographs of the gold-coated oleogel recorded at different magnifications: (**a**) 100×, (**b**) 200×, (**c**) 400× and (**d**) 800×. The images reveal a compact and homogeneous surface morphology characterized by rounded microdomains, radial crystalline-like structures, and folded lamellar regions associated with the self-assembled gel network.

Representative SEM micrographs of the gold-coated oleogel are presented in Figure 9. At low magnification (Figure 9a,b), the oleogel exhibited a relatively continuous and homogeneous surface morphology without visible macroscopic cracks or phase separation. The matrix appeared compact, suggesting efficient immobilization of the oil phase within the gel network.

Increasing the magnification revealed the presence of numerous rounded and irregularly shaped microdomains distributed throughout the surface (Figure 9c,d). These structures were surrounded by folded and radially organized regions, generating a heterogeneous but interconnected architecture. Such morphological features are commonly associated with the self-assembly and crystallization processes occurring during oleogel formation.

As observed in Figure 9c, several circular domains displaying radial organization could be distinguished. These structures resemble crystalline aggregates or spherulitic-like domains generated during gelator crystallization [46]. Similar morphologies have been reported for oleogels structured through crystalline gelators, where crystal growth and aggregation contribute to the development of a three-dimensional network capable of entrapping the liquid oil phase [8,47].

The higher-magnification image shown in Figure 9d further highlights the wrinkled and lamellar appearance of the surface. The presence of these folded structures suggests the formation of interconnected crystalline regions that may contribute to the mechanical stability and rheological behavior of the oleogel [48].

No evidence of extensive pore formation, phase segregation, or structural collapse was observed throughout the analyzed areas, indicating good structural integrity of the formulation. The compact morphology observed by SEM is consistent with the formation of a stable oleogel network capable of maintaining the oil phase within the gel matrix.

Overall, the SEM investigation demonstrated that the oleogel possesses a continuous microstructured architecture characterized by crystalline-like domains embedded within a compact matrix, supporting the formation of a stable three-dimensional network.

#### 2.3.8. Total Phenolic Content of the Oleogel

The total phenolic content (TPC) of the developed oleogel was determined using the Folin–Ciocalteu assay and expressed as gallic acid equivalents (GAE). The formulation contained 0.246 ± 0.008 mg GAE/g oleogel (245.7 ± 8.0 μg GAE/g), corresponding to 24.57 ± 0.80 mg GAE/100 g of product. This result confirms the successful incorporation of reducing phytoconstituents into the lipid-based formulation.

The measured TPC exceeded the value expected from the chamomile hydroglyceric extract alone, indicating that the overall reducing capacity of the oleogel also reflects the contribution of other formulation components. In particular, sea buckthorn oil contains naturally occurring phenolic compounds together with other antioxidant constituents, while tocopherol and additional reducing substances present in the lipid matrix may also contribute to the response of the Folin–Ciocalteu reagent.

Therefore, the reported TPC should be interpreted as the total reducing capacity of the oleogel, expressed as gallic acid equivalents, rather than as the concentration of phenolic compounds originating exclusively from the chamomile extract. The quantified TPC was subsequently used as the reference value for calculating the cumulative release of phenolic compounds in the Franz diffusion cell study.

### 2.4. Functional Properties of the Oleogel

#### 2.4.1. Occlusivity Test

The occlusive properties of the developed oleogel were evaluated using an *in vitro* gravimetric water-loss assay over a 48 h period. To determine the specific contribution of the oleogel matrix, the blank oleogel (without chamomile extract) was included as an additional control, while white petrolatum served as the reference occlusive formulation. As shown in Table 5, the blank oleogel exhibited the highest occlusion factors, followed by the chamomile-loaded oleogel. In comparison, white petrolatum showed the lowest occlusion factors at both evaluation time points. The occlusion factor decreased over time for all formulations; however, both oleogel formulations maintained superior barrier properties throughout the study.

The higher occlusion factors observed for the blank oleogel indicate that the structured lipid network of the oleogel matrix was primarily responsible for limiting water evaporation. Incorporation of chamomile extract produced only a slight reduction in the occlusive effect, suggesting a minor influence on the structural organization of the gel matrix without compromising its barrier-forming capacity. Nevertheless, both oleogel formulations exhibited superior occlusive performance compared with white petrolatum, demonstrating an enhanced ability to reduce water evaporation. This behavior may be attributed to the formation of a continuous lipid film promoted by the structured oleogel network, which effectively limits water loss and enhances moisture retention.

The observed occlusive effect is consistent with previous reports demonstrating the importance of lipid barriers in limiting water loss from the skin surface. Agren and Wijesinghe reported that highly occlusive dressings reduced normal transepidermal water loss by approximately 70% and promoted water retention within the stratum corneum, highlighting the beneficial role of occlusion in maintaining skin hydration [49]. Furthermore, Lukić et al. reported occlusion factors ranging from approximately 57% to 81% after 24 h and from 57% to 77% after 48 h for creams containing lipid and ethanolic extracts obtained from wheat, maize, and sunflower by-products [50]. The occlusion factors obtained in the present study are therefore within the range reported for natural extract-based topical formulations with documented moisturizing and barrier-enhancing properties. Differences between studies may be attributed to variations in formulation composition, lipid phase organization, and experimental conditions employed for occlusivity assessment.

The pronounced occlusive properties observed in the developed oleogel may represent an additional advantage over conventional chamomile creams or emulgels, as the lipid-rich continuous phase can reduce transepidermal water loss while supporting skin barrier restoration.

Taken together, these findings suggest that the developed formulation possesses favorable barrier-forming properties and may contribute to improved skin hydration, making it a promising candidate for topical applications requiring prolonged moisturization and skin protection.

#### 2.4.2. *In Vitro* Release of Total Phenolic Compounds

**Figure 10:**
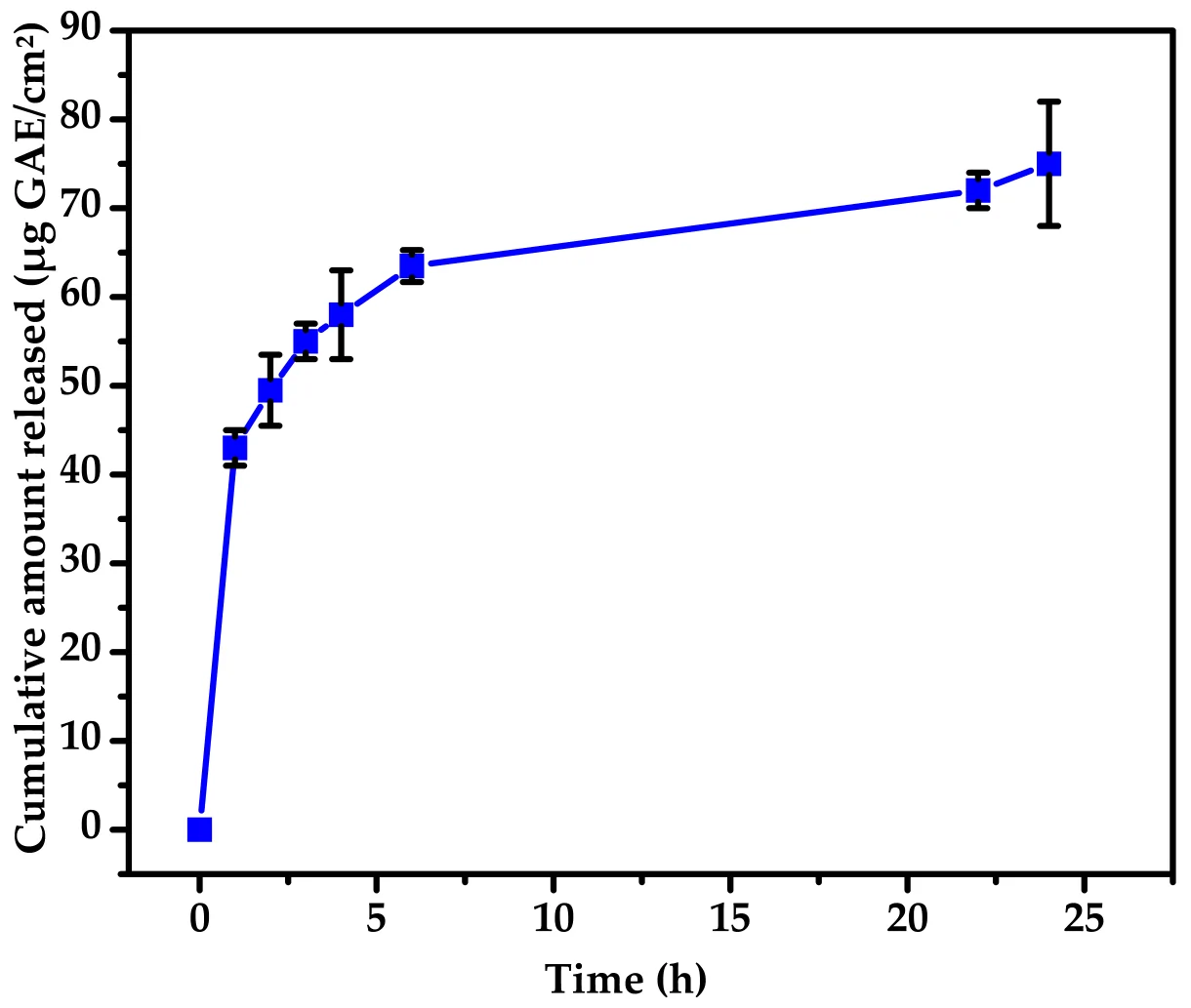
*In vitro* release profile of total phenolic compounds from the developed oleogel determined using Franz diffusion cells. Results are expressed as cumulative amount released per unit diffusion area (μg GAE/cm^2^, mean ± SD, n = 3).

The *in vitro* release of total phenolic compounds from the developed oleogel was evaluated using Franz diffusion cells and expressed as gallic acid equivalents (GAE). The cumulative release profile exhibited a biphasic behavior, characterized by an initial burst release followed by a slower diffusion-controlled phase (Figure 10). The individual release profiles obtained from the three independent Franz diffusion cells are presented in Figure S10 (Supplementary Materials).

After 1 h, 37.05 ± 1.66% of the total phenolic content incorporated into the donor compartment had diffused through the membrane. The released fraction increased progressively to 42.57 ± 3.48% after 2 h, 54.51 ± 1.55% after 6 h, and reached 64.97 ± 5.92% after 24 h. When expressed per unit diffusion area, the cumulative amount released increased from 42.94 ± 1.92 μg GAE/cm^2^ at 1 h to 63.17 ± 1.80 μg GAE/cm^2^ after 6 h and 75.29 ± 6.86 μg GAE/cm^2^ after 24 h.

The observed release behavior was also influenced by the selected lipid phase, in which sweet almond and sea buckthorn oils acted as reservoirs for the incorporated phytoconstituents, while the beeswax-structured network limited their diffusion, thereby contributing to the sustained release profile.

The pronounced initial release is most likely associated with phenolic compounds located near the oleogel surface or within the more accessible hydrophilic domains of the formulation, allowing their rapid partitioning into the receptor medium. Subsequently, the release rate gradually decreased, indicating diffusion of phenolic compounds from the interior of the structured oleogel matrix, where mass transport is restricted by the three-dimensional lipid network. At the end of the experiment, approximately 35% of the initial phenolic content remained associated with the oleogel matrix, suggesting partial retention of bioactive compounds within the lipid phase. Such behavior is considered advantageous for topical formulations because it may contribute to prolonged local availability of antioxidant constituents following application.

To further characterize the release behavior, the experimental release data were analyzed using several mathematical kinetic models, including the zero-order, first-order, Higuchi, Korsmeyer–Peppas and Hixson–Crowell models. The corresponding kinetic parameters are summarized in Table 6. The cumulative amount of phenolic compounds released per unit diffusion area was also plotted against the square root of time according to the Higuchi model (Figure S11, Supplementary Materials).

Among the investigated models, the Korsmeyer–Peppas model provided the highest coefficient of determination (R^2^ = 0.998), followed by the Higuchi model (R^2^ = 0.916), whereas the zero-order, first-order and Hixson–Crowell models showed comparatively lower goodness-of-fit (Table 6).

The Higuchi release constant was K = 7.12 ± 1.58 μg GAE·cm^−2^·h^−1^/^2^, supporting diffusion as the predominant release mechanism. The low release exponent obtained from the Korsmeyer–Peppas model (n = 0.22), together with the comparatively good fit of the Higuchi model, is consistent with the experimentally observed biphasic release profile, comprising an initial burst release followed by a slower diffusion-governed phase. Overall, the mathematical kinetic modeling provides additional mechanistic support for the experimentally observed release behavior of the developed oleogel.

Taken together, these findings demonstrate that the developed oleogel provides a controlled biphasic release of phenolic compounds, characterized by an initial burst release followed by a diffusion-controlled phase, thereby ensuring sustained local availability of the incorporated phytoconstituents after topical application.

**Figure 11:**
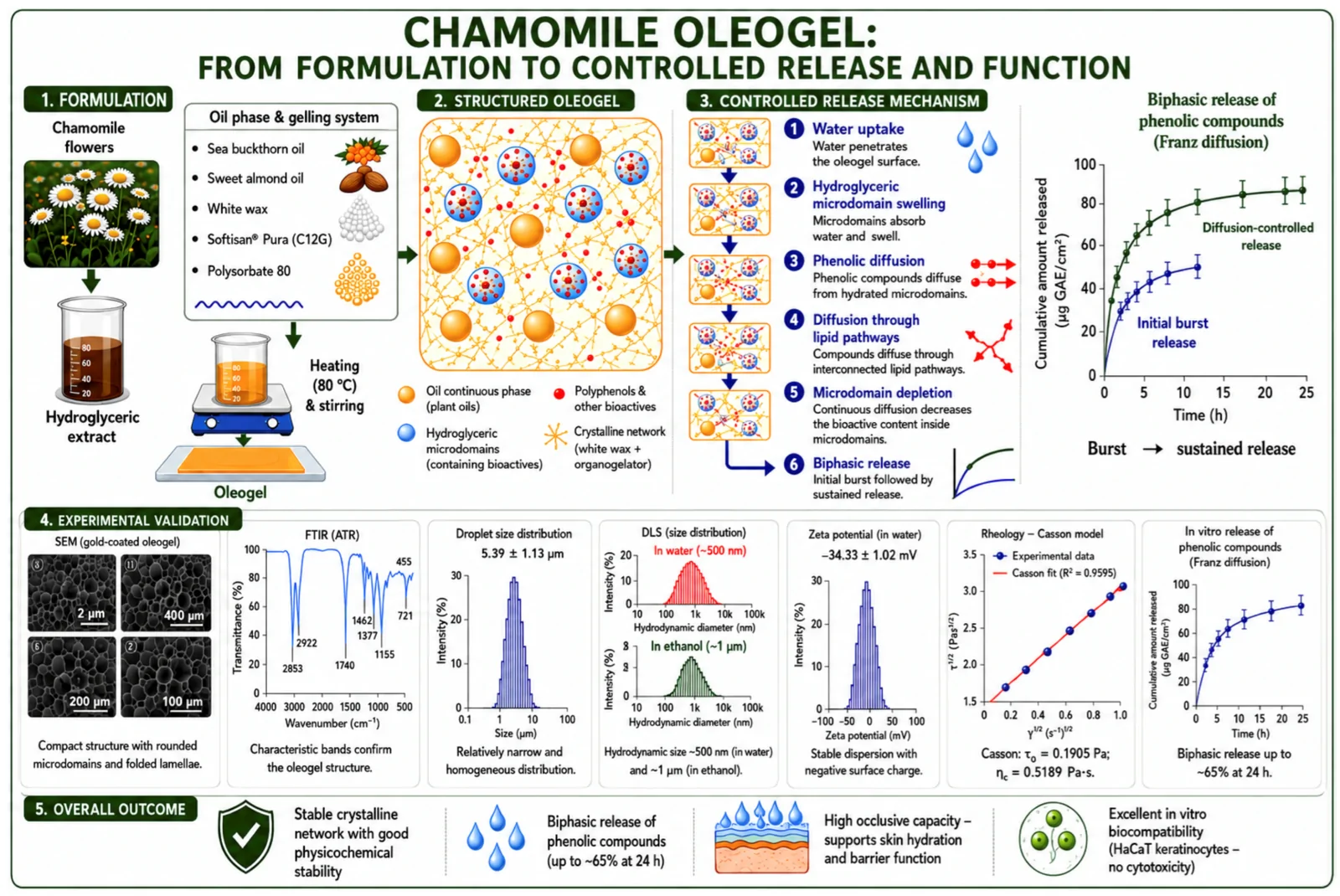
Schematic illustration of the formulation, structural organization, controlled release mechanism, and physicochemical characterization of the bioactive chamomile oleogel. Created in BioRender. Mihailescu, C. M. (2026) https://BioRender.com/a2xko9t (accessed on 31 July 2026).

To provide an integrated overview of the developed system, a schematic illustration summarizing the formulation process, structural organization, controlled release mechanism, physicochemical characterization, and overall performance of the bioactive chamomile oleogel is presented in Figure 11. The schematic integrates the principal experimental findings and illustrates the relationship between the oleogel microstructure, its physicochemical properties, and the controlled biphasic release of phenolic compounds.

### 2.5. Antioxidant Activity of Oleogel

**Figure 12:**
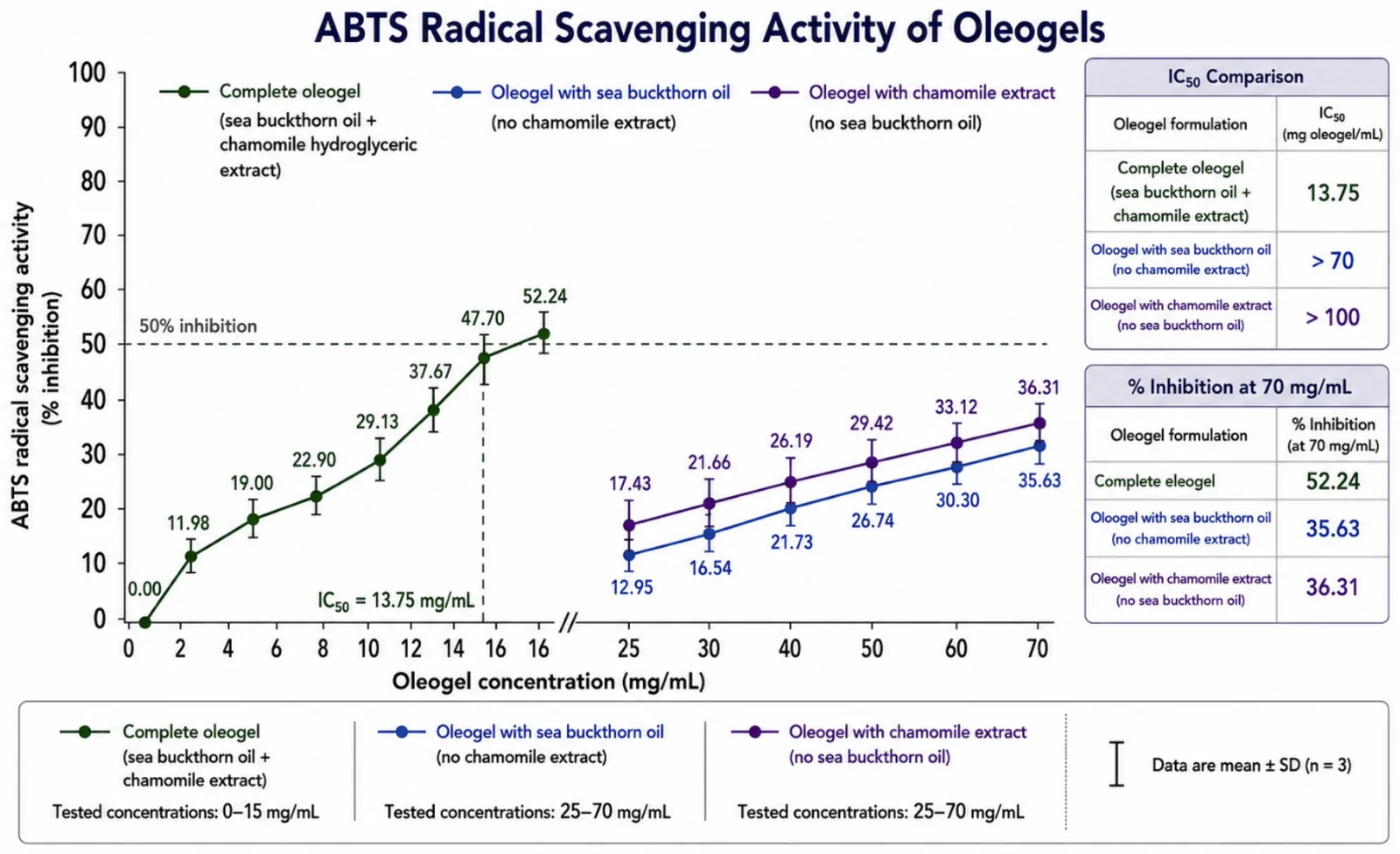
Comparison of the ABTS radical scavenging activity of the complete oleogel and the two control formulations.

The antioxidant activity of the complete oleogel and the two control formulations was evaluated using the ABTS radical scavenging assay to assess the contributions of chamomile hydroglyceric extract and sea buckthorn oil (Figure 12).

The complete oleogel exhibited the highest antioxidant activity, with an IC_50_ value of 13.75 mg oleogel/mL. Two control formulations were also investigated: an oleogel containing sea buckthorn oil in which the chamomile extract was replaced by the corresponding hydroglyceric vehicle, and an oleogel containing chamomile extract in which sea buckthorn oil was replaced by an equivalent amount of sweet almond oil.

Both controls showed concentration-dependent radical scavenging activity. For the sea buckthorn oil-containing control, inhibition increased from 12.95% at 2.5 mg/mL to 35.63% at 70 mg/mL, whereas the chamomile extract-containing control produced inhibition values ranging from 17.43% at 25 mg/mL to 40.00% at 100 mg/mL. As neither formulation reached 50% inhibition, their IC_50_ values were considered to exceed 70 mg/mL and 100 mg/mL, respectively.

Within the common concentration range of 25–70 mg/mL, the two control oleogels displayed comparable antioxidant profiles, reaching 35.63% and 36.31% inhibition at 70 mg/mL. The substantially greater activity of the complete oleogel suggests that the simultaneous presence of hydrophilic phenolic compounds from chamomile extract and lipophilic antioxidants from sea buckthorn oil provides a more effective radical scavenging system than either component alone.

### 2.6. Biocompatibility Assessment on Keratinocytes

HaCaT cells, an immortalized human keratinocyte cell line, represent a well-established *in vitro* model for evaluating the biocompatibility and cytotoxicity of dermatological formulations due to their preserved proliferative, differentiation, and regenerative properties. Since keratinocytes play a fundamental role in skin repair and re-epithelialization, maintaining their viability is essential for the development of safe and effective topical therapeutic systems.

**Figure 13:**
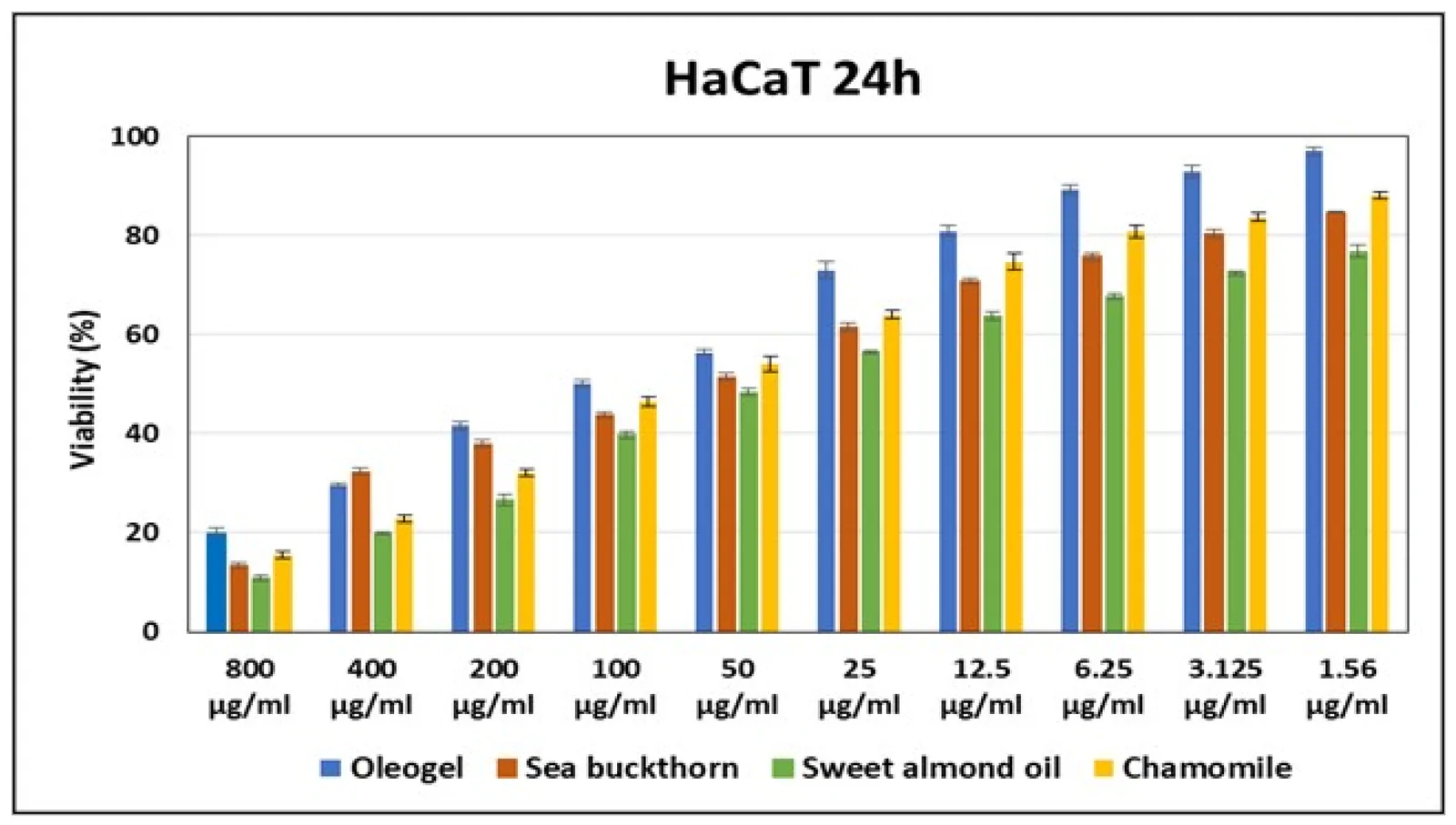
Effect of the tested formulations on the viability of the human keratinocyte cell line HaCaT. Cells were exposed to concentrations ranging from 1.56 to 800 μg/mL for 24 h. Untreated cells were used as a control and considered to have 100% viability. Results are expressed as mean ± standard deviation (SD), obtained from three independent experiments (n = 3).

**Figure 14:**
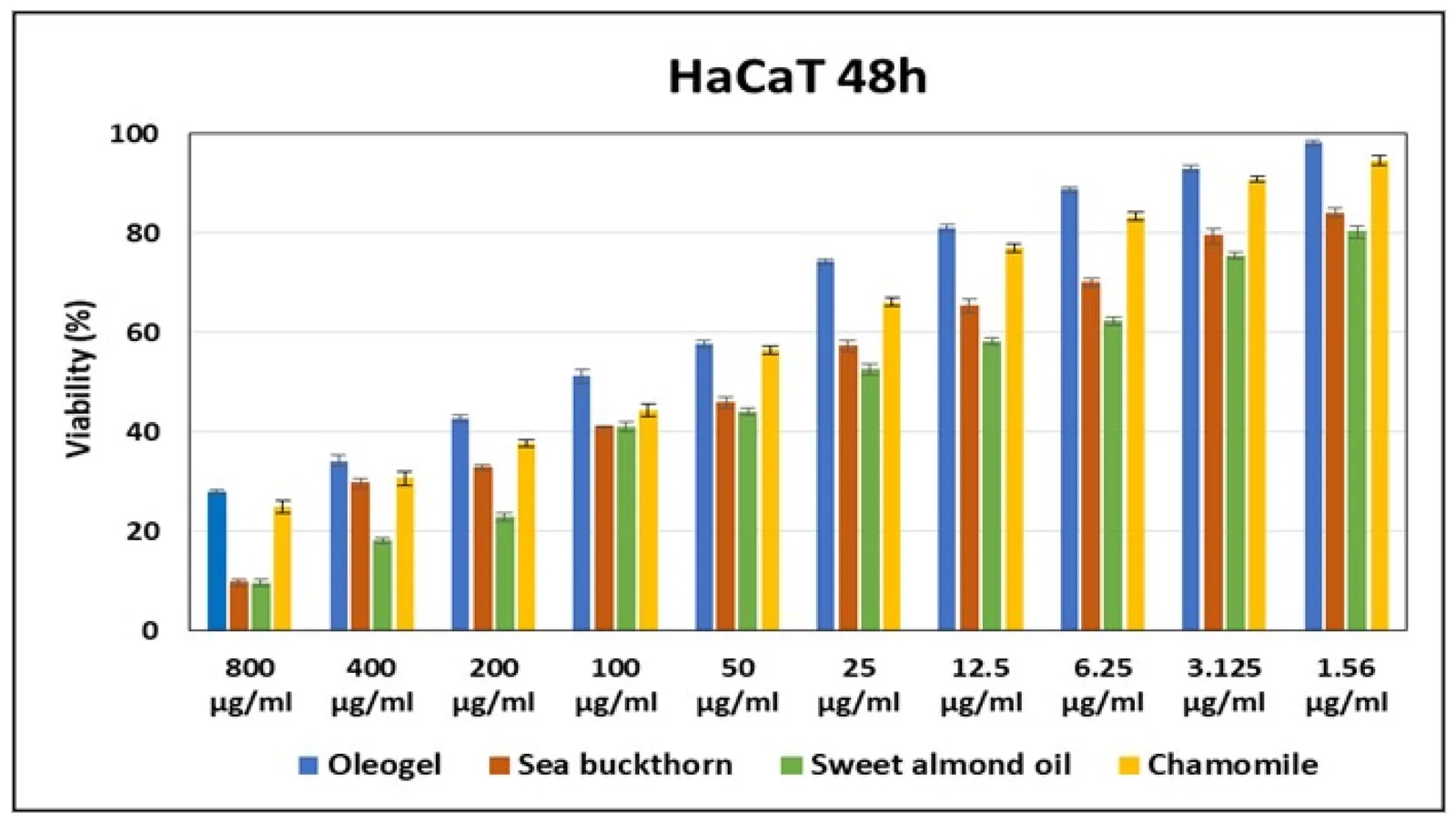
Effect of the tested formulations on the viability of the human keratinocyte cell line HaCaT. Cells were exposed to concentrations ranging from 1.56 to 800 μg/mL for 48 h. Untreated cells were used as a control and considered to have 100% viability. Results are expressed as mean ± standard deviation (SD), obtained from three independent experiments (n = 3).

The cytotoxicity of the investigated formulations was assessed by measuring HaCaT cell viability following exposure to concentrations ranging from 1.56 to 800 μg/mL for 24 and 48 h. The results demonstrated a clear concentration-dependent response for all formulations, with cell viability progressively increasing as the concentration decreased, indicating that cytotoxic effects were dose dependent (Figure 13 and Figure 14).

Among the tested formulations, the oleogel consistently exhibited the highest level of biocompatibility at both incubation times. It maintained superior cell viability across the entire concentration range and showed particularly favorable performance at lower concentrations, where viability approached that of the untreated control. This enhanced compatibility is likely related to the three-dimensional gel network, which enables a controlled release of bioactive compounds and reduces direct cellular exposure to high concentrations.

The sea buckthorn oil formulation displayed intermediate biocompatibility, with reduced viability at higher concentrations but good cellular compatibility under diluted conditions. These effects may be associated with its high content of biologically active compounds, including carotenoids, unsaturated fatty acids, tocopherols, and phytosterols, which provide antioxidant and regenerative benefits but may induce metabolic stress when present at elevated concentrations.

The chamomile formulation also demonstrated an intermediate cytotoxicity profile, with higher cell viability than sea buckthorn and almond oil at most concentrations. Its favorable behavior at low concentrations can be attributed to the antioxidant and anti-inflammatory properties of chamomile phenolic compounds and flavonoids, although slight cytotoxic effects were observed at higher doses.

In contrast, the sweet almond oil formulation showed the lowest cell viability throughout most of the tested concentration range, indicating reduced cellular tolerance under the experimental conditions. Nevertheless, viability increased substantially at lower concentrations, confirming that its cytotoxicity was also dose dependent and suggesting that safe application is achievable through appropriate concentration optimization.

According to ISO 10993-5 criteria, all formulations were considered non-cytotoxic at sufficiently low concentrations, as they maintained cell viability above the 70% threshold [51].

Overall, the findings indicate that the oleogel formulation provides the most favorable balance between biocompatibility and controlled delivery of bioactive compounds. Its ability to preserve keratinocyte metabolic activity supports its potential use as a promising delivery platform for wound healing, skin regeneration, and other dermatological and biomedical applications.

## 3. Conclusions

This study demonstrates that a hydroglyceric chamomile extract can be successfully incorporated into a structured lipid-based oleogel while preserving the functional properties of its phenolic constituents. The developed formulation combines the antioxidant potential of plant-derived bioactive compounds with the favorable physicochemical characteristics of an oleogel, resulting in a stable, homogeneous, and skin-compatible semisolid system.

Comprehensive characterization confirmed appropriate organoleptic properties, pH, rheological behavior, and stability under accelerated storage conditions. Complementary structural investigations by dynamic light scattering, zeta potential analysis, FTIR spectroscopy, and scanning electron microscopy further demonstrated the formation of a well-organized lipid network with preserved chemical integrity, compact microstructure, and good colloidal stability.

Phytochemical analysis verified the presence of characteristic phenolic acids and flavonoids in the chamomile extract and confirmed their successful incorporation into the oleogel. The formulation retained measurable antioxidant activity after processing, indicating that the preparation procedure did not compromise the functionality of the incorporated phytoconstituents. Moreover, the Franz diffusion study revealed a biphasic release profile, characterized by an initial burst release followed by sustained diffusion of phenolic compounds through the lipid matrix.

The combined physicochemical, rheological, release, occlusive, antioxidant and biocompatibility results indicate that the developed oleogel represents a promising multifunctional platform for topical delivery of plant-derived bioactive compounds. Compared with conventional chamomile creams or emulgels, the anhydrous oleogel system offers improved physicochemical stability, enhanced occlusive properties, reduced dependence on preservatives, and the potential for sustained release of phenolic compounds. Although the present study demonstrated excellent physical and rheological stability during accelerated storage, the long-term chemical stability of the incorporated bioactive compounds was not investigated and represents a limitation of the current work. Therefore, future studies should focus on the quantitative monitoring of key phytochemical markers during prolonged storage under different temperature conditions, together with ex vivo skin permeation studies, disease-relevant skin models, in vivo efficacy evaluation, and long-term stability under real storage conditions, to establish a comprehensive stability and performance profile of the formulation.

## 4. Materials and Methods

### 4.1. Chemicals and Plant Materials

All solvents and reagents used in this study were of analytical grade. Ethanol, potassium hydroxide, potassium iodide, iodine monochloride, potassium persulfate, glacial acetic acid, gallic acid, sodium carbonate, and phosphate-buffered saline (PBS, pH 7.4) were purchased from Sigma-Aldrich (St. Louis, MO, USA). Sodium thiosulfate, hydrochloric acid, glycerol, chloroform, 2,2′-azino-bis(3-ethylbenzothiazoline-6-sulfonic acid) (ABTS), Folin–Ciocalteu Reagent, diethyl ether, phenolphthalein, and polysorbate 80 were obtained from Merck (Darmstadt, Germany). Sea buckthorn oil was obtained from Hofigal (Bucharest, Romania), while sweet almond oil, vitamin E, white petrolatum, and white wax were purchased from Elemental (Oradea, Romania). Softigen Pura and Softisan 649 were supplied by Elton (Elton Corporation S.A., Bucharest, Romania). Purified water obtained from a Milli-Q purification system (Merck Millipore, Burlington, MA, USA) was used for the preparation of all solutions, extracts, and formulations throughout the study.

*Matricaria chamomilla* L. (chamomile) flowers were collected in June 2025 from Dumitrești village (Vrancea County, Romania), air-dried, and stored under appropriate conditions. The plant material was identified according to taxonomic criteria in the Department of Botany, Faculty of Pharmacy from Titu Maiorescu University, Bucharest.

The hydroglyceric extract of chamomile flowers was prepared by mixing finely powdered dried plant material with a glycerol–water mixture (40:60, *w*/*w*) at a plant material concentration of 10% (*w*/*w*). The mixture was subjected to ultrasound-assisted extraction for 1 h using an ultrasonic bath (Elmasonic S 50 R, Elma Schmidbauer GmbH, Singen, Germany) operating at 37 kHz and an effective ultrasonic power of 150 W. The resulting extract was filtered through Whatman No. 1 filter paper and stored at 4 °C until further use.

### 4.2. Characterization of the Natural Oils Used as the Oleogel Base

#### 4.2.1. Physicochemical Quality Assessment of Sea Buckthorn Oil and Sweet Almond Oil

The physicochemical characterization included the determination of refractive index, relative density, acid value, saponification value, iodine value and peroxide value, in accordance with standardized methods (AOAC and ISO) [52]. All analyses were performed in triplicate at room temperature using properly calibrated equipment to ensure accuracy and reproducibility of the results.

- Refractive index

The refractive index of the vegetable oil samples were determined using an Abbe refractometer calibrated with distilled water (n = 1.3330 at 20 °C) [53]. Measurements were performed at 20 ± 0.2 °C under identical experimental conditions to ensure accuracy and reproducibility.

- Relative density

The relative density of the vegetable oils was determined by the pycnometric method using a calibrated 25 mL pycnometer [54]. Measurements were performed at 20 ± 0.2 °C and the relative density was calculated as the ratio of the oil mass of an equal volume of distilled water.

- Acid value

The acid value was determined by titration according with standard methods [55]. Briefly, 2.0 g of oil sample was dissolved in a neutralized ethanol-diethyl ether mixture (1:1, *v*/*v*) and titrated with 0.1 N ethanolic KOH using phenolphthalein as indicator. The acid value was calculated according to equation:

(1) $$Acid\;value=\frac{V\cdot N\cdot 56.1}{W},$$

where V is the volume of KOH used (mL), N is the normality of KOH, W is the weight of the oil sample (g).

- Saponification value

The saponification value was determined by reflux titration according to standard methods [56]. Briefly, 2.0 g of oil sample was refluxed with 0.5 N ethanolic KOH for 60 min, and the excess alkali was titrated with 0.5 N HCl using phenolphthalein as the indicator. A blank determination was analyzed under the conditions. The saponification value was calculated using the formula:

(2) $$Saponification\;value=\frac{\left(B-S\right)\cdot N\cdot 56.1}{W},$$

where B is the volume of HCl used for the blank (mL), S is the volume used for the sample (mL), N is the normality of HCl, W is the weight of the oil sample (g).

- Iodine value

The iodine value was determined using the Wijs method according to standard procedures [57]. Briefly, 0.3 g of oil sample was reacted with Wijs reagent in the dark for 30 min, followed by the addition of potassium iodide and distilled water. The liberated iodine was titrated with 0.1 N sodium thiosulfate solution using starch as an indicator. A blank was analyzed under identical conditions. The iodine value was calculated using the formula:

(3) $$Iodine\;value=\frac{\left(B-S\right)\cdot N\cdot 12.69}{W},$$

where B is the volume of Na_2_S_2_O_3_ used for the blank (mL), S is the volume of Na_2_S_2_O_3_ used for the sample (mL), N is the normality of Na_2_S_2_O_3_ solution, 12.69 conversion factor to grams of iodine, W is the weight of the oil sample (g).

- Peroxide value

The peroxide value was determined by the iodometric titration method in accordance with standard procedures [58]. Briefly, approximately 5 g of oil sample was dissolved in an acetic acid-chloroform mixture, treated with potassium iodide and the liberated iodine was titrated with 0.01 N sodium thiosulfate using starch as an indicator. A blank was analyzed under identical conditions. The peroxide value (meq O_2_/kg oil) was calculated using the formula:

(4) $$Peroxide\;value=\frac{\left(S-B\right)\cdot N\cdot 1000}{W},$$

where S is the volume of Na_2_S_2_O_3_ used for the sample (mL), B is the volume of Na_2_S_2_O_3_ used for the blank (mL), N is the normality of Na_2_S_2_O_3_ solution, 1000 is the conversion factor that converts grams of oil to kilograms, W is the weight of the oil sample (g).

#### 4.2.2. GC-MS Analysis of Sea Buckthorn Oil and Sweet Almond Oil

The fatty acid composition of sea buckthorn oil and sweet almond oil was determined by gas chromatography–mass spectrometry (GC–MS) after conversion to fatty acid methyl esters (FAMEs). Briefly, 0.1 g of each oil was dissolved in 5.0 mL petroleum ether, followed by the addition of 50 mL of 0.5 M methanolic hydrochloric acid. Acid-catalyzed transesterification was performed under reflux at approximately 65 °C for 1 h with continuous stirring. After cooling, the FAMEs were extracted with isooctane, washed with distilled water to neutral pH, dried over anhydrous sodium sulfate, filtered, and subjected to GC–MS analysis.

Analyses were carried out on a Thermo Scientific GC–MS system (Thermo Fisher Scientific, Waltham, MA, USA) equipped with a TG-WAX MS capillary column (30 m × 0.25 mm × 0.25 μm). Helium was used as the carrier gas at a flow rate of 1.5 mL/min. Samples (1 μL) were injected in split mode (1:333). Fatty acids were identified by comparison of their retention times and mass spectra with the NIST Tandem Mass Spectral Library and reference retention data. The relative abundance of each fatty acid was expressed as the percentage of the total peak area. GC–MS analysis was used for the qualitative identification and semi-quantitative characterization of the oil samples [59,60].

### 4.3. Phytochemical Characterization of the Chamomile Hydroglyceric Extract

#### 4.3.1. HPLC Analysis of Chamomile Hydroglyceric Extract

The phytochemical profile of the chamomile hydroglyceric extract was evaluated by high-performance liquid chromatography with diode-array detection (HPLC–DAD) using an Agilent 1260 Infinity system (Agilent Technologies, Santa Clara, CA, USA). Separation was achieved on a C18 reversed-phase column (250 × 4.6 mm, 5 μm) maintained at 30 °C. The mobile phase consisted of 0.1% phosphoric acid and an acetonitrile–methanol mixture, delivered under gradient elution over 70 min at a flow rate of 1.0 mL/min. The injection volume was 5 μL. Chromatograms were recorded at 230 and 330 nm, while UV spectra were acquired between 200 and 400 nm.

The chromatographic method was used for the identification and relative quantification of the major phenolic compounds and flavonoids present in the extract [61]. The HPLC–DAD analysis was performed to obtain a qualitative phytochemical profile, whereas quantitative standardization using individual marker compounds was beyond the scope of the present study and will be addressed in future investigations to further improve batch-to-batch reproducibility.

#### 4.3.2. Total Polyphenolic Assay of Chamomile Hydroglyceric Extract

The total polyphenol content of the chamomile hydroglyceric extract was determined by the Folin–Ciocalteu spectrophotometric method using gallic acid as the calibration standard [59]. Briefly, 1 mL of extract was mixed with 2.5 mL of dilluted Folin–Ciocalteu reagent, followed by 2 mL of sodium carbonate solution and 4.5 mL of deionized water. The mixture was incubated at room temperature for 60 min before measuring the absorbance at 765 nm with a UV–Vis spectrophotometer (UV-6300 PC, VWR International, Vienna, Austria).

Polyphenol concentration was calculated from the gallic acid calibration curve (R^2^ = 0.9997), using a reagent blank for baseline correction. Measurements were performed in triplicate, and the results are reported as mean ± standard deviation.

#### 4.3.3. Determination of Antioxidant Activity by the ABTS Assay

The antioxidant capacity of the chamomile hydroglyceric extract and the developed oleogel formulations was assessed using the ABTS radical cation decolorization assay as previously described, with slight modifications [60]. The ABTS•^+^ working solution was prepared by combining equal volumes of 7 mM ABTS and 2.45 mM potassium persulfate and allowing the mixture to stand in the dark at room temperature for 15 h. Prior to analysis, the solution was diluted with ethanol to an absorbance of 0.70 ± 0.02 at 734 nm.

The chamomile hydroglyceric extract was diluted with ethanol to obtain the required concentration range. For oleogel analysis, 1 g of sample was dispersed in 10 mL ethanol under vigorous stirring to extract the antioxidant constituents, after which appropriate dilutions of the ethanolic extract were prepared.

The ABTS radical scavenging assay was performed under identical experimental conditions for the complete oleogel and for both control formulation.

For each determination, 0.5 mL of sample solution was mixed with 4.5 mL of the ABTS•^+^ working solution. Following incubation for 6 min in the dark at room temperature, absorbance was measured at 734 nm using a UV–Vis spectrophotometer (UV-6300 PC, VWR International, Vienna, Austria), with ethanol serving as the blank. All analyses were carried out in triplicate.

Radical scavenging activity was calculated according to Equation (5):

(5) $$ABTS\;scavenging\;activity\;\left(\%\right)=\frac{A_{control}-A_{sample}}{A_{control}}\times 100,$$

where A_control_ represent the absorbance of the control and A_sample_, the absorbance solutions, respectively.

The IC_50_ value, defined as the concentration required to reduce ABTS•^+^ radical activity by 50%, was estimated from the concentration–response curve. Data are presented as mean ± standard deviation (SD).

### 4.4. Formulation Composition and Preparation of the Oleogel

The oleogel was formulated using the ingredients listed in Table 7 (per 100 g).

The oleogel was prepared using the melting method. The oil phase consisted of caprylic/capric triglycerides (Softigen^®^Pura), bis-diglyceryl polyacyladipate-2 (Softisan^®^649), sweet almond oil, and white wax as the organogelator. All oil phase components, except for thermosensitive ingredients, were accurately weighed and transferred into a glass beaker. The mixture was heated to 80–85 °C under continuous magnetic stirring until complete melting of the white wax and formation of a clear, homogenous solution. The system was maintained at this temperature for several minutes to ensure uniform dispersion of the gelator. Subsequently, the formulation was allowed to cool under continuous stirring. At approximately 45 °C, sea buckthorn oil, hydroglycerinic chamomile extract, Polisorbate 80, and tocopherol were incorporated into the mixture to ensure uniform dispersion of the polar phase within the lipid matrix, while avoiding thermal degradation of sensitive compounds. Any additional lipophilic active ingredients or fragrance were also added at this stage. The resulting mixture was gently stirred to ensure uniform distribution and then poured into suitable containers. The oleogel was allowed to cool at room temperature until complete gelation occurred.

The final product was a homogeneous, semi-solid oleogel with good spreadability and stability. Although the formulation contained a minor dispersed polar phase in the form of a hydroglyceric chamomile extract, it was classified as an oleogel because the continuous phase remained lipid-based and the semisolid structure was primarily governed by the three-dimensional crystalline network formed by white wax. Polysorbate 80 facilitated the homogeneous dispersion and stabilization of the polar phase within the structured lipid matrix. Accordingly, the formulation was considered a structured lipid-based oleogel containing a minor dispersed polar phase, in agreement with the broader definition of oleogels reported in the recent literature [3,43].

To evaluate the individual contribution of the bioactive ingredients, two control oleogels were prepared following the same procedure as the complete formulation. In the first control, the chamomile hydroglyceric extract was replaced with an equivalent amount of the corresponding hydroglyceric vehicle while maintaining the overall formulation composition. In the second control, sea buckthorn oil was replaced by an equivalent amount of sweet almond oil to preserve the lipid phase ratio. All subsequent procedures for preparation, homogenization, and storage were identical to those used for the complete oleogel.

### 4.5. Oleogel Characterization

The physicochemical and structural characterization of oleogels is essential for understanding their performance in topical applications. Rheological behavior, spreadability, and zeta potential provide key insights into the internal structure, applicability, and stability of the system, supporting the evaluation of its structure–function relationship.

#### 4.5.1. Organoleptic Evaluation

The organoleptic characteristic of the developed oleogel, including appearance, color, odor, texture and homogeneity, were systematically evaluated by a panel of five trained assessors. Each attribute was scored using a five-point scoring system (1 = poor, 5 = excellent). All evaluations were conducted under standardized conditions at room temperature, ensuring consistent lighting and environmental parameters [61]. Each attribute was qualitatively assessed using predefined descriptive criteria, allowing for a comprehensive characterization of the formulation’s sensory profile. Particular attention was given to parameters such as structural uniformity, surface smoothness, and olfactory acceptability. The organoleptic analysis provided essential insights into the sensory performance, stability, and overall user acceptability of the oleogel, contributing to the evaluation of its suitability for topical application.

#### 4.5.2. pH Determination

Given the anhydrous nature of the oleogel, pH evaluation was performed on a 10% (*w*/*v*) aqueous dispersion prepared by dispersing 1 g of sample in 10 mL of distilled water. The pH was measured at room temperature using a calibrated pH meter (Consort, Turnhout, Belgium). All measurements were performed in triplicate (n = 3), and the results are presented as mean ± standard deviation.

#### 4.5.3. Accelerated Stability Assessment

##### Centrifugation Test

The physical stability of the oleogel was evaluated by centrifugation. Two samples (5 g each) were centrifuged at 3000 rpm for 30 min, at room temperature (25 ± 2 °C), using a Micro 220R benchtop centrifuge (Hettich Zentrifugen GmbH, Tuttlingen, Germany). Following centrifugation, the samples were visually inspected for signs of phase separation, oil bleeding, sedimentation, or structural disruption [62].

##### Thermal Stress Test

The oleogel samples were stored at different temperatures (4 °C, 25 °C and 37 °C) for an initial period of 30 days (Table S9, Supplementary Materials), but the monitoring is extended for 3 months. Samples were periodically evaluated for changes in color, consistency and phase separation [63,64].

##### Freeze–Thaw Cycles

The formulation was subject to five freeze–thaw cycles [64], alternating between—20 °C and 30 °C, with each cycle lasting 24 h. This range is commonly used to evaluate the physical stability of semi-solid formulations, as low temperatures may induce lipid crystallization, while subsequent return to ambient conditions can leads to structural reorganization. The freeze–thaw cycles were carried out using a UV75i incubator (Memmert, Germany) for the thawing stage and an ULUF 400 ultra-low temperature freezer (Arktiko, Denmark) for the freezing stage.

After each cycle, the samples were examined for phase separation, texture changes and network destabilization.

#### 4.5.4. Droplet Size

The droplet size of the oleogel was determined using a Motic digital imaging system (Motic Microscopes, Kowloon, Hong Kong). A small aliquot of the oleogel was placed on a glass slide, covered with a coverslip, and observed under different magnifications. The diameters of 100 droplets were measured using a calibrated eyepiece scale, and the mean droplet size and size distribution were calculated [23,65].

The polydispersity index (PDI) was calculated using the relation (SD/mean)^2^, where SD represents the standard deviation and mean the average droplet diameter, based on the experimentally obtained size distribution data.

#### 4.5.5. Rheological Characterization

##### Viscosity and Flow Behavior

The rheological properties of the oleogel were evaluated using a FungiLab Smart rotational viscometer (FungiLab S.A., Barcelona, Spain) equipped with spindle No. 7. Measurements were performed at room temperature at different rotational speeds (100, 150, and 200 rpm) to assess the flow behavior of the system.

Samples were carefully introduced into the measuring system to avoid air incorporation, and viscosity values were recorded after stabilization at each rotational speed. All measurements were carried out at predetermined time intervals (T0, T7, T14, T21, T30, T56 and T84) to monitor the evolution of the rheological properties during storage.

The results were expressed as viscosity (mPa·s) and reported as mean ± SEM based on triplicate determinations. This approach allowed the evaluation of both the shear-dependent behavior and the storage stability of the oleogel over time [35].

##### Yield Stress

In addition to viscosity measurements yield stress of the oleogel was investigated using a controlled shear-rate protocol. The shear rate was progressively decreased from 0.75 to 0.20 s^−1^, in six discrete steps, with a stabilization period of 60 s at each step to allow the sample to reach a steady-state response before the corresponding shear stress was recorded. The corresponding shear stress and viscosity values were automatically recorded by the instrument.

The experimental data were fitted using the Casson rheological model, a two-parameter model commonly applied to viscoplastic, shear-thinning systems exhibiting a yield stress, according to the following equation:

(6) *√τ* = *√τ*_0_ + *√ηc*·*√γ̇*,

where τ is the shear stress, γ̇ is the shear rate, τ_0_ represents the Casson yield stress, and ηc is the Casson plastic viscosity. The Casson parameters were obtained from the linearized form of the equation by linear regression analysis. The coefficient of determination (R^2^) was used to evaluate the quality of the model fitting [52].

It should be noted that the shear rate range investigated with the rotational spindle viscometer (0.20–0.75 s^−1^) was constrained by the torque calibration limits of the spindle/instrument combination for this structured, yield-stress formulation, and therefore spans less than one decade. Consequently, the reported Casson parameters (τ_0_, ηc) should be regarded as descriptive of the low-shear, near-rest structural behavior of the oleogel rather than as a validated full-range flow curve. Future studies will employ a cone-and-plate or parallel-plate rheometer to extend the characterization across a broader shear-rate window (1–2 decades or more), enabling a more comprehensive validation of the yield-stress model.

##### Structural Recovery

The structural recovery behavior of the oleogel was evaluated using a three-interval thixotropy-inspired test (3-ITT) performed using a FungiLab Smart rotational viscometer [66].

During the first interval, the sample was subjected to a low rotational speed (20 rpm) for 2 min to determine the initial viscosity ($\eta_{0}$). In the second interval, the rotational speed was increased to 200 rpm for 5 min in order to disrupt the oleogel network and obtain the viscosity after shear-induced breakdown ($\eta_{breakdown}$). Subsequently, the rotational speed was reduced to 20 rpm and the recovery of the system was monitored for 10 min. Viscosity values were recorded after 1, 3, 5 and 10 min of recovery.

Structural recovery (%) was calculated as the ratio between the recovered viscosity and the initial recovery, using the following formula:

(7) $$Structural\;recovery\;(\%)=\frac{\eta_{10}}{\eta_{0}}\times 100,$$

where $\eta_{0}$ represent the initial viscosity, and $\eta_{10}$ represent the recovered viscosity after 10 min.

Viscosity loss (%) was determined from the decrease in viscosity after the high-shear stage with the equation:

(8) $$Viscosity\;loss\;\left(\%\right)=\frac{\eta_{0}-\eta_{breakdown}}{\eta_{0}}\times \;100,$$

where η_0_ is the initial viscosity measured before the application of high shear, and η_breakdown_ is the viscosity recorded during the high-shear stage.

Recovery efficiency (%) was calculated to evaluate the extent of structural rebuilding after shear cessation, with the formula:

(9) $$Recovery\;efficiency\;(\%)=\frac{\eta_{10}-\eta_{breakdown}}{\eta_{0}-\eta_{breakdown}}\times \;100,$$

where η_0_ represents the initial viscosity of the oleogel, η_breakdown_ represents the viscosity measured during the breakdown stage under high shear, and η_10_ represents the viscosity recorded after 10 min of the recovery stage.

#### 4.5.6. Spreadability

The spreadability of the oleogel was assessed using the Ojeda Arbussa method [55]. For the analysis, 1 g of the oleogel was placed between two glass plates (20 × 20 cm) for 1 min. The initial diameter of the spread sample was recorded. A standardized weight of 125 g was then applied, followed by the sequential addition of extra weights (10, 20, 30, 50, 100, 150, 200 and 250 g) at 1 min intervals. After each weight increment, the diameter of the spread area was measured. The results were expressed as the surface area of the spread, calculated as a function of the applied mass according to the following equation:

(10) $$S_{i}=\frac{\pi \cdot {di}^{2}}{4},$$

where S_i_ (mm^2^) is the spreading area obtained under the applied mass i (g), d_i_ is the mean diameter (mm) reached by the sample. Experiments were performed in triplicate (n = 3), and the results were expressed as mean ± SEM.

#### 4.5.7. Structural Characterization

##### Dynamic Light Scattering (DLS) and Zeta Potential Analysis

The hydrodynamic size distribution and surface charge characteristics of the oleogel formulation were evaluated using a Delsa™ Nano C Particle Analyzer (Beckman Coulter, Brea, CA, USA), operating based on Dynamic Light Scattering (DLS) and Electrophoretic Light Scattering (ELS) principles.

For sample preparation, 0.1 g of oleogel was dispersed in 30 mL of solvent under gentle stirring to obtain a sufficiently diluted suspension suitable for light scattering measurements. Analyses were performed using both ultrapure water and ethanol as dispersion media in order to investigate the influence of solvent polarity on the colloidal behavior of the dispersed oleogel structures.

Hydrodynamic diameter measurements were performed at 25 °C using the DLS mode of the instrument, and the results were expressed as particle size distribution profiles and mean hydrodynamic diameter values [67]. Due to the semi-solid nature of the oleogel system, the measured dimensions should be interpreted as apparent hydrodynamic sizes corresponding to dispersed structural domains, aggregates, and solvent-swollen assemblies rather than individual particles.

Zeta potential measurements were carried out using the electrophoretic mobility mode of the instrument at 25 °C. The obtained zeta potential values were used to assess the electrostatic stability of the dispersed oleogel structures in the investigated media. All measurements were performed in triplicate, and the results were reported as mean values ± standard deviation.

Considering the complex and heterogeneous composition of oleogels, which may contain crystalline domains, self-assembled gelator structures, and dispersed oil-rich regions, both DLS and zeta potential results were interpreted as complementary indicators of colloidal behavior and dispersion stability rather than absolute particle characterization parameters.

##### Fourier Transform Infrared Spectroscopy (FTIR)

Fourier transform infrared spectroscopy (FTIR) was employed to investigate the chemical structure of the oleogel formulation and to evaluate potential interactions between the formulation components and the silicon substrate. FTIR spectra were recorded using a Bruker Tensor 27 spectrometer (Bruker Optics, Ettlingen, Germany) equipped with a platinum ATR accessory with diamond crystal. Spectra were acquired in the range of 4000–400 cm^−1^ with a spectral resolution of 4 cm^−1^, averaging 64 scans per sample.

Two samples were analyzed: the bulk oleogel formulation and the oleogel deposited onto a silicon substrate. Prior to analysis, samples were allowed to equilibrate at room temperature. The obtained spectra were processed using the instrument software and interpreted according to characteristic vibrational bands reported in the literature for lipid-based systems, triglycerides, fatty acid esters, waxes, and plant-derived constituents.

##### Scanning Electron Microscopy (SEM)

Electron microscopy was performed using a FEI Nova NanoSEM 630 field-emission scanning electron microscope (FEI Company, Hillsboro, OR, USA). SEM imaging was performed at an accelerating voltage of 5 kV, an emission current of 198 μA, a gun pressure of 1.81 × 10^−7^ Pa, and a chamber pressure of 3.4 × 10^−3^ Pa.

The oleogel samples were mounted on aluminum stubs using conductive carbon adhesive tape. To minimize charging effects during electron beam exposure, the samples were sputter-coated with a thin gold (Au) layer (approximately 5–10 nm) using an SPI-Module™ Sputter Coater (Structure Probe, Inc., West Chester, PA, USA) operated at a plasma current of 18 mA for 40 s under an argon atmosphere at a working pressure of approximately 1–2 mbar and a target-to-sample distance of 50 mm. SEM micrographs were acquired under high-vacuum conditions at magnifications ranging from 100× to 800× to evaluate the overall morphology, surface organization, and microstructural characteristics of the oleogel network.

### 4.6. Functional Properties of the Oleogel

#### 4.6.1. Occlusivity Test

The occlusive properties of the chamomile-loaded oleogel formulation and the corresponding blank oleogel were evaluated using an *in vitro* gravimetric method based on water evaporation reduction [47,68]. Briefly, glass beakers containing a fixed volume of distilled water (25 g), were covered with Whatman glass microfiber filter (9.0 cm), and sealed to prevent lateral water loss. A defined amount of the each formulation was uniformly spread over the surface of the filter paper to form a continuous film. White petrolatum served as the reference occlusive formulation, while a beaker without any applied formulation served as the control.

The prepared systems were stored at room temperature for 48 h, and their weights were recorded at the beginning of the experiment and after the incubation period. The water loss was calculated from the difference in mass, and the occlusion factor (F) was determined using the equation:

(11) $$F=\frac{A-B}{A}\;x\;100,$$

where *A* is s the amount of water loss from the control and *B* is the amount of water loss from the test formulation. Higher F values indicate greater occlusive properties. Each determination was carried out in triplicate, and the results were reported as mean ± standard deviation. Statistical analysis was performed using one-way analysis of variance (ANOVA), followed by Tukey’s multiple comparison test to evaluate differences among the blank oleogel, chamomile-loaded oleogel, and white petrolatum at each evaluation time point. Differences were considered statistically significant at *p* < 0.05.

#### 4.6.2. *In Vitro* Release of Total Polyphenolic Compounds Using Franz Diffusion Cell

The *in vitro* release of total phenolic compounds from the developed oleogel was evaluated using vertical Franz diffusion cells. An accurately weighed amount of oleogel (370 mg) was uniformly spread over the donor compartment. A cellulose acetate membrane served as the diffusion barrier between the donor and receptor compartments. The effective diffusion area was 0.785 cm^2^, corresponding to a membrane diameter of 1.0 cm.

The receptor compartment (8 mL) was filled with a hydroalcoholic medium consisting of phosphate buffer (pH 7.4) and ethanol (80:20, *v*/*v*) in order to maintain sink conditions throughout the experiment. The receptor phase was continuously stirred at 600 rpm. Aliquots of 1 mL were withdrawn after 1, 2, 3, 4, 6, 22 and 24 h and immediately replaced with an equal volume of fresh receptor medium to maintain a constant receptor volume.

The phenolic content in the receptor samples was determined as described in Section 2.3.8 and expressed as gallic acid equivalents (GAE). Cumulative release values were corrected for the sampling volume according to equation:

(12) $$Q_{n}=C_{n}\cdot V_{r}+V_{s}\cdot \sum_{i=1}^{n-1}C_{i},$$

where $Q_{n}$ is the cumulative amount of phenolic compounds transferred into the receptor compartment at time n, expressed as μg GAE; $C_{n}$ is the concentration measured in the receptor phase at time n, expressed as μg GAE/mL; $V_{r}\;\mathrm{is}$ the receptor compartment volume, equal to 8 mL vs. is the sampling volume, equal to 1 mL.

$\sum_{i=1}^{n-1}C_{i}$ represents the sum of the concentrations measured in all previous samples.

The cumulative amount of phenolic compounds released per unit diffusion area was calculated according to equation:

(13) $$Q_{area}=\frac{Q_{n}}{A},$$

and expressed as: $g\;GAE/cm^{2}$.

To further characterize the release mechanism, the cumulative amount of phenolic compounds released per unit diffusion area was plotted as a function of the square root of time according to the Higuchi diffusion model [69]. The apparent release constant (K) was calculated from the slope of the linear regression.

All experiments were performed in triplicate using three independent Franz diffusion cells, and the results are expressed as mean ± standard deviation (SD).

### 4.7. Biocompatibility Assessment on Keratinocytes

The immortalized human keratinocyte cell line HaCaT was obtained from Cell Line Service GmbH (Catalogue No. 330493, Eppelheim, Germany). Cells were routinely maintained in Dulbecco’s Modified Eagle’s Medium (DMEM) supplemented with 10% fetal bovine serum (FBS), 2 mM L-glutamine, and antibiotics (100 U/mL penicillin and 100 μg/mL streptomycin, Sigma-Aldrich, St. Louis, MO, USA). Cell cultures were incubated at 37 °C in a humidified atmosphere containing 5% CO_2_. Upon reaching approximately 60% confluence (24 h after seeding), the cells were treated with the tested formulations at the specified concentrations for the indicated exposure periods. Following treatment, adherent cells were detached using a non-enzymatic PBS solution supplemented with 1 mM EDTA, washed twice with phosphate-buffered saline (PBS), and subsequently processed for cytotoxicity evaluation. Untreated cells maintained under identical culture conditions served as the negative control throughout the study.

Cell viability was determined in triplicate using the CellTiter 96^®^ Aqueous One Solution Cell Proliferation Assay (Promega, Madison, WI, USA), according to the manufacturer’s instructions. The assay, performed in flat-bottom 96-well plates (Falcon, Corning, NY, USA), is based on the reduction of the MTS tetrazolium compound by metabolically active cells to a soluble formazan product, the amount of which is directly proportional to the number of viable cells. The reagent contains MTS [3-(4,5-dimethylthiazol-2-yl)-5-(3-carboxymethoxyphenyl)-2-(4-sulfophenyl)-2H-tetrazolium, inner salt] and phenazine ethosulfate (PES), which acts as an electron-coupling reagent to enhance formazan formation.

For each experiment, HaCaT cells were seeded into flat-bottom 96-well plates at a density of 1 × 10^4^ cells/well in 100 μL of complete culture medium and incubated for 24 h to allow cell attachment. Subsequently, the culture medium was replaced with fresh medium containing increasing concentrations of the tested formulations, and the cells were incubated for either 24 or 48 h. At the end of the treatment period, 20 μL of CellTiter 96^®^ Aqueous One Solution reagent (MTS/PES) was added to each well, followed by incubation for 4 h at 37 °C under standard culture conditions with intermittent gentle shaking to facilitate formazan formation. The absorbance of the resulting soluble formazan product, which is directly proportional to cellular metabolic activity and viability, was measured at 492 nm using a DYNEX Technologies microplate reader (MRS; DYNEX Technologies, Chantilly, VA, USA).

Cell viability was expressed as the percentage of viable cells relative to untreated control cultures, which were considered to represent 100% viability, and was calculated according to the following equation:

(14) $$Cell\;viability\left(\%\right)=\frac{absorbance\;of\;treated\;cells-absorbance\;of\;culture\;medium}{absorbance\;of\;untreated\;cells-absorbance\;of\;culture\;medium}\times 100\;,$$

The mean ± standard deviation (SD) of experiments performed in triplicate was used to calculate the percentage of viability relative to untreated cells [70,71].

## Figures and Tables

**Table 1:** Physicochemical parameters of sea buckthorn and sweet almond oils.

Parameter	Reference Range	Sea Buckthorn Oil (SBO) (Mean ± SD)	Sweet Almond Oil (SAO) (Mean ± SD)
Refractive index (20 °C)	1.465–1.475 1.460–1.470	1.469 ± 0.001	1.468 ± 0.001
Relative density (20 °C)	0.900–0.925 0.910–0.915	0.924 ± 0.001	0.915 ± 0.001
Acid value (mg KOH/g)	≤10 ≤0.5	3.2 ± 0.2	0.4 ± 0.1
Saponification value (mg KOH/g)	175–195 190–200	182 ± 2	190 ± 2
Iodine value (g I_2_/100 g)	60–90 90–110	85 ± 3	93 ± 2
Peroxide value (meq O_2_/kg)	≤10 ≤5	5.8 ± 0.4	3.2 ± 0.3

**Table 2:** Compound s identified by GC–MS in sea buckthorn oil and sweet almond oil.

No	Compound	Retention Time (min)	Sea Buckthorn Oil Relative Area (%)	Sweet Almond Oil Relative Area (%)
1.	Palmitic acid	9.742	35.06	4.76
2.	Palmitoleic acid	10.242	39.17	-
3.	Methyl stearate	14.020	-	2.86
4.	Oleic acid	14.370	4.32	65.10
5.	cis-Vaccenic acid	14.479	5.13	-
6.	Linoleic acid	15.296	16.31	27.28

**Table 3:** Phenolic compounds identified in the chamomile hydroglyceric extract by HPLC-DAD analysis.

Compound	Retention Time (RT) (min)	Area (mAU·min)	Relative Area (%)
Protocatechuic acid *	3.40	4.516	2.57
Caffeic acid *	4.20	9.921	5.65
Tannin derivative	5.01	4.631	2.64
Chlorogenic acid	14.00	6.042	3.44
p-Coumaric acid	16.09	49.508	28.20
Quercetin derivative	27.03	3.404	1.94
Unidentified phenolic compound	28.80	51.547	29.36
Rutin hydrate	39.07	5.451	3.11
Apigenin-7-O-glucoside	44.83	33.801	19.25
Quercetin	48.72	6.733	3.84
Total	—	175.554	100.00

* Tentative identification based on retention time and UV spectral characteristics at 280 nm.

**Table 4:** The organoleptic characteristics of the oleogel.

Characteristic	Oleogel	Initial	After 30 Days
		Score (Mean ± SD)	Score (Mean ± SD)
Appearance	Semi-solid, cohesive system; maintains shape after application	4.9 ± 0.1	4.8 ± 0.2
Color	Intensive yellow-orange, uniform, characteristic of sea buckthorn oil	5.0 ± 0.0	4.9 ± 0.1
Odor	Mild, characteristic odor of natural oils, no rancid notes	4.7 ± 0.3	4.6 ± 0.3
Homogeneity	Uniform structure, no visible phase separation or oil extrudation	5.0 ± 0.06	5.0 ± 0.09
Texture	Smooth, slightly firm, non-gritty, easy to spread	4.8 ± 0.3	4.8 ± 0.3
Consistency	Moderately firm with good structural integrity	4.9 ± 0.2	4.9 ± 0.2
Film-forming ability	Easy to apply, form continuous film on surface	4.9 ± 0.3	4.9 ± 0.3

**Table 5:** Occlusion factor (%) of the blank oleogel, the chamomile-loaded oleogel, and white petrolatum after 24 and 48 h. Values are expressed as mean ± SD (n = 3). Statistical analysis was performed using one-way ANOVA followed by Tukey’s multiple comparison test. Different superscript letters within the same column indicate statistically significant differences (*p* < 0.05).

Formulation	Occlusion Factor (%)—24 h	Occlusion Factor (%)—48 h
Blank oleogel	68.89 ± 0.41 ^a^	59.02 ± 0.28 ^a^
Chamomile-loaded oleogel	67.95 ± 0.39 ^a^	56.45 ± 0.26 ^b^
White petrolatum	64.86 ± 0.32 ^b^	44.78 ± 0.35 ^c^

**Table 6:** Kinetic parameters obtained by fitting mathematical models to the mean cumulative release profile of total phenolic compounds from the oleogel (n = 3 independent Franz diffusion cells).

Model	Linear Form (y vs. x)	R^2^	Rate Constant/Exponent
Zero-order	Q vs. t	0.830	K_0_ = 0.94% h^−1^
First-order	ln(100 − Q) vs. t	0.884	K_1_ = 0.020 h^−1^
Higuchi	Q vs. √t	0.916	K_H = 6.14% h^−0^·^5^
Korsmeyer–Peppas (≤60%)	log(M_t_/M∞) vs. log t	0.998	n = 0.22; k = 0.37
Hixson–Crowell	[M∞^⅓ − (M∞ − Q)^⅓] vs. t	0.867	K_s = 0.024 h^−1^

Fitted to the seven measured time points (theoretical origin excluded); Korsmeyer–Peppas fitted to the five points with ≤60% release. Q = cumulative amount released; M∞ = total releasable amount.

**Table 7:** Composition of the developed bioactive oleogel and functional role of each ingredient.

Ingredient	% (*w*/*w*)	Role in Formulation
Sea buckthorn oil	7.5	Bioactive emollient oil rich in carotenoids; provides antioxidant, regenerative, and skin-conditioning effects.
Sweet almond oil	6.0	Emollient; improves spreadability and skin feel.
Glyceryl Stearate SE	5.0	Co-emulsifier and consistency agent; stabilizes the formulation and enhances viscosity and structural integrity.
Caprylic/Capric Triglyceride (and) Hydrogenated Rapeseed oil *	33.0	Primary emollient base; enhances skin feel, improves spreadability and acts as a petrolatum natural alternative
Bis-Diglyceryl Polyacyladipate-2 **	31.0	Occlusive emollient and moisturizer, with high stability against rancidity (lanolin alternative)
*Chamomilla matricaria* hydroglyceric extract	7.5	Humectant and soothing agent; provides anti-inflammatory and skin-calming effects
White wax	6.0	Structuring agent: forms a semi-solid network and increases formulation consistency and stability
Polysorbate 80 ***	3.0	Nonionic surfactant, improves dispersion of components and stabilizes the system
Tocopheryl acetate ****	0.5	Antioxidant and oxidative stability enhancer

* Softigen Pura; ** Softisan 649; *** Tween 80; **** Vitamin E.

## Data Availability

The original contributions presented in this study are included in the article/Supplementary Materials. Further inquiries can be directed to the corresponding authors.

## Supplementary Materials

The following supporting information can be downloaded at: https://www.mdpi.com/article/10.3390/gels12080704/s1, Figure S1: GC-MS chromatogram of Sea Buckthorn Oil.; Figure S2: GC-MS chromatogram of Sweet Almond Oil; Figure S3: HPLC chromatogram of *Matricaria chamomilla* hydroglyceric extract; Figure S4: Macroscopic appearance of the developed oleogel, showing its homogeneous structure and characteristic yellow-orange coloration attributed to the presence of sea buckthorn oil; Figure S5: Radar chart illustrating the sensory profile of the developed oleogel based on organoleptic evaluation. The numerical scores for each parameter are shown on the chart. Scores were assigned using a 5-point scale, where 1 indicates very poor and 5 indicates excellent performance; Figure S6: Radar chart illustrating the sensory profile of the developed oleogel based on organoleptic evaluation. The numerical scores for each parameter are shown on the chart. Scores were assigned using a 5-point scale, where 1 indicates very poor and 5 indicates excellent performance; Figure S7: Zeta potential distribution of the oleogel dispersed in distilled water; Figure S8: Zeta potential distribution of the oleogel dispersed in ethanol; Figure S9: ATR–FTIR spectrum of the oleogel deposited on a silicon substrate; Figure S10: Individual release profiles of total phenolic compounds obtained from three independent Franz diffusion cells; Figure S11: Higuchi plot showing the relationship between the cumulative amount of phenolic compounds released per unit diffusion area (μg GAE/cm^2^) and the square root of time; Table S1: The pH values for oleogel; Table S2: Effect of storage temperature on the physical stability of the oleogel; Table S3: Freeze–thaw stability of the oleogel; Table S4: Rheological tests of the oleogel; Table S5: Experimental values used for Casson plot construction (Figure 4), obtained from rotational rheological measurements of the oleogel formulation; Table S6: Structural recovery profile of the oleogel (mean ± SD, n = 3); Table S7: Structural recovery parameters of the oleogel determined by the three interval thixotropy test (3 ITT); Table S8: The results of spreadability for the oleogel; Table S9: Thermal stress conditions of oleogel.
