# Supplementary material for: A Bioactive Oleogel Incorporating Chamomile Hydroglyceric Extract: Physicochemical Characterization, Occlusive Properties, Controlled Release and Biocompatibility
Source: Gels. 2026 Aug 6;12(8):704. doi: 10.3390/gels12080704 (PMC13512593; doi:10.3390/gels12080704)
Supplement: Supplementary file 1 [file gels-12-00704-s001.zip › gels-4434996-supplementary.pdf]

Supplementary Materials for: "A Bioactive Oleogel Incorporating Chamomile Hydroglyceric Extract: Physicochemical Characterization, Occlusive Properties, Controlled Release and Biocompatibility"

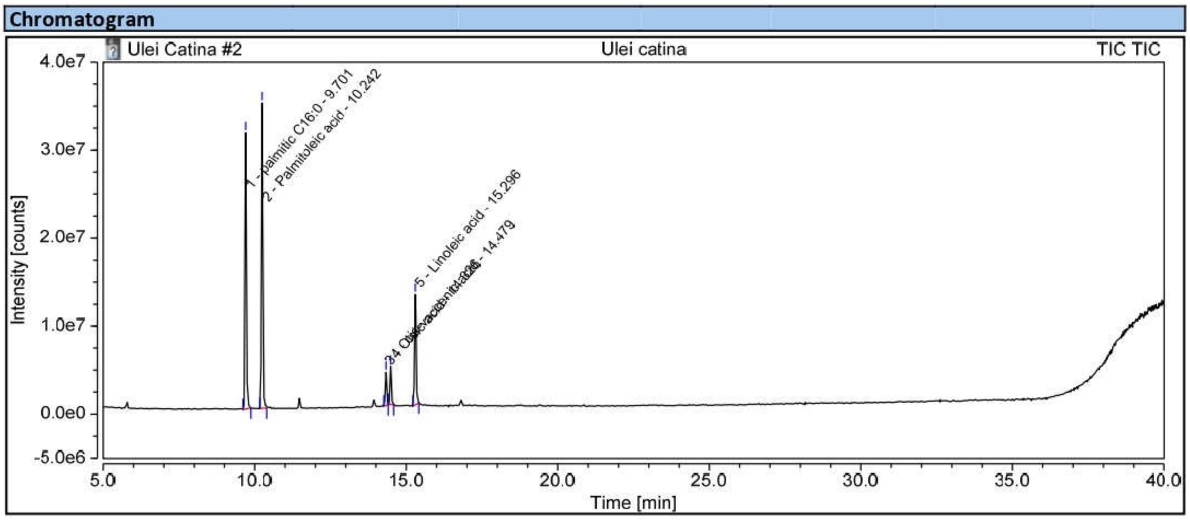

Figure S1. GC-MS chromatogram of Sea Buckthorn Oil.

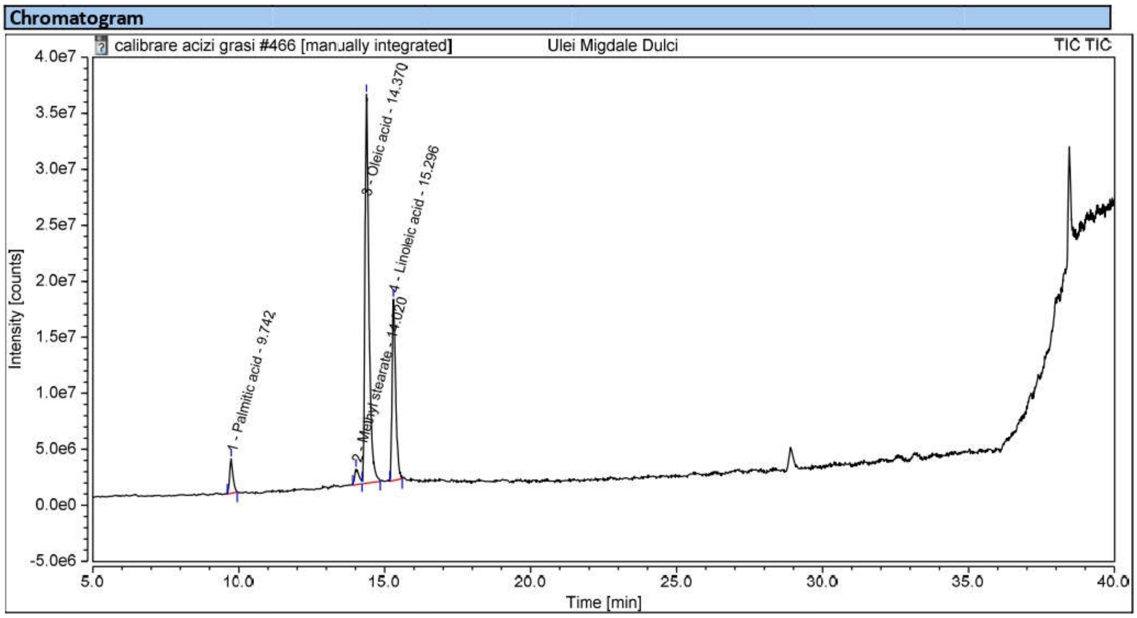

Figure S2. GC-MS chromatogram of Sweet Almond Oil.

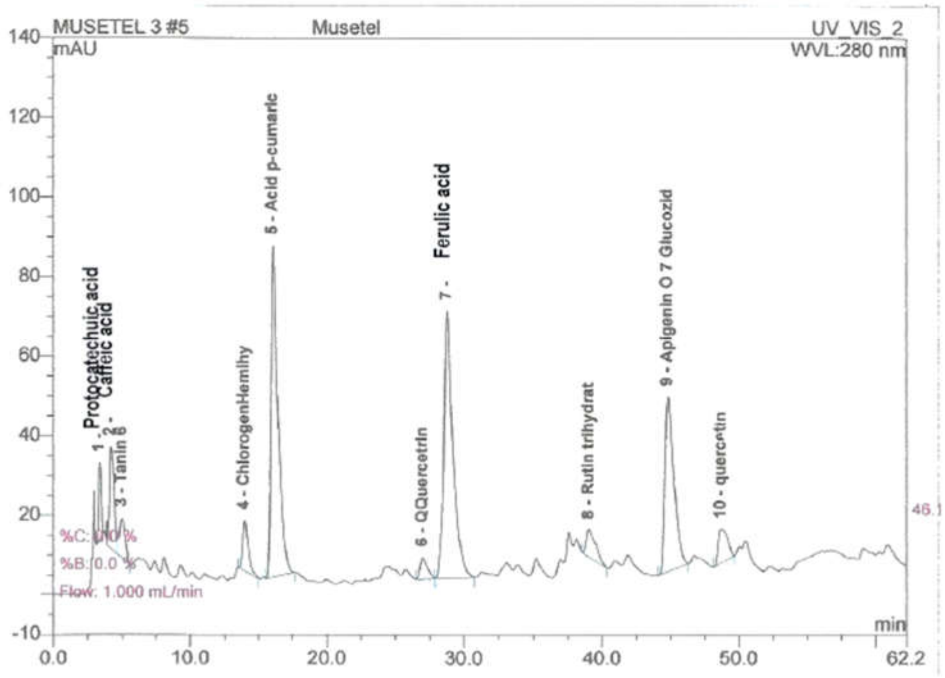

Figure S3. HPLC chromatogram of *Matricaria chamomilla* hydroglyceric extract.

Detailed: HPLC-DAD analysis revealed a complex phenolic profile of the chamomile hydroglyceric extract, comprising phenolic acids and flavonoids (Figure S3, Supplementary Material; Table 4). As summarized in Table 3, p-coumaric acid (28.20%) and apigenin-7-O-glucoside (19.25%) were the major identified compounds, while chlorogenic acid, rutin hydrate, quercetin, and several minor phenolic constituents were also detected. An additional chromatographic peak, representing 29.36% of the total peak area, remained unidentified but exhibited UV spectral characteristics consistent with a phenolic compound.

Two early-eluting peaks were tentatively assigned to protocatechuic acid and caffeic acid based on their retention behavior and UV absorption characteristics at 280 nm. The predominance of phenolic acids and flavonoids agrees with the phytochemical profile previously reported for *Matricaria chamomilla* and provides a phytochemical basis for the antioxidant and skin-protective properties expected from the hydroglyceric extract. These bioactive constituents are likely to contribute to the functional performance of the developed oleogel intended for topical application.

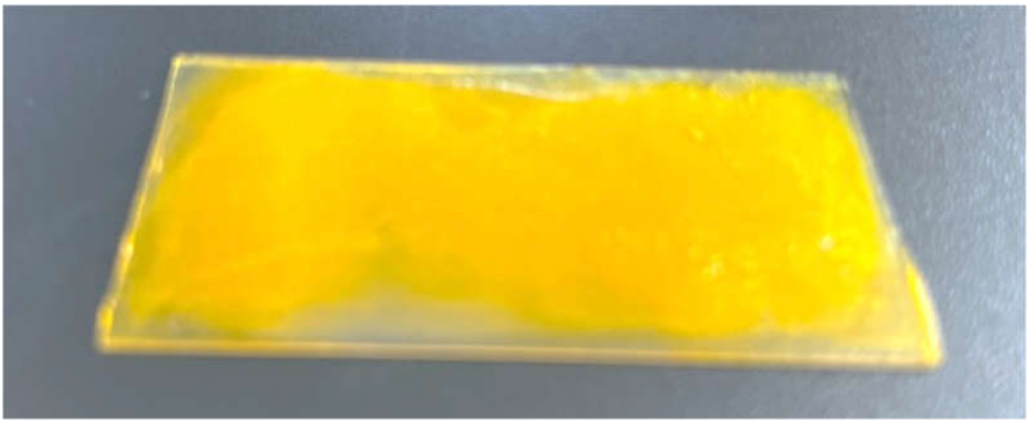

Figure S4. Macroscopic appearance of the developed oleogel, showing its homogeneous structure and characteristic yellow-orange coloration attributed to the presence of sea buckthorn oil.

Detailed organoleptic evaluation:
The oleogel exhibited a uniform structure with no visible phase separation, confirming its physical stability over the evaluation period. As illustrated in Figure 4, from the Supplementary Material, the intense yellow-orange color, attributed to the presence of sea buckthorn oil, remained stable and homogenous, contributing to the product's visual appeal. Texture and spreadability showed consistently high values, reflecting a smooth, cohesive system with good application properties and ease of distribution on the skin. Slightly lower scores were observed for odor, which may be associated with the intrinsic characteristics of the lipid components, however, it remained within an acceptable and pleasant range.

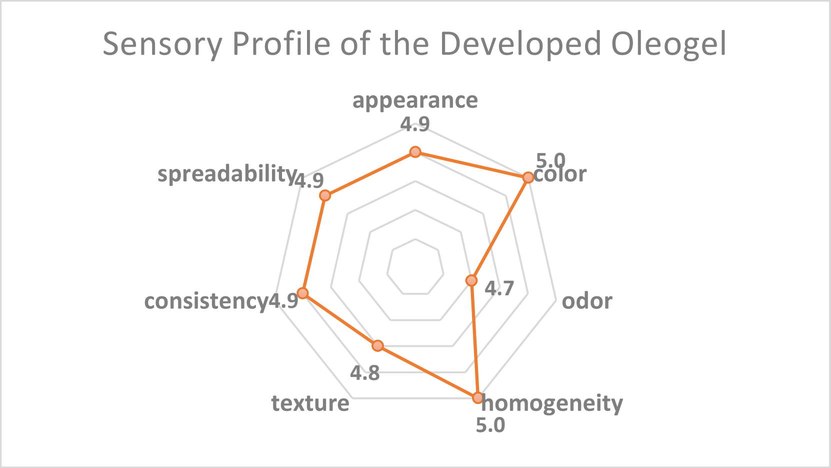

Figure S5. Radar chart illustrating the sensory profile of the developed oleogel based on organoleptic evaluation. The numerical scores for each parameter are shown on the chart. Scores were assigned using a 5-point scale, where 1 indicates very poor and 5 indicates excellent performance.

Table S1. The pH values for oleogel.

pH values (mean±SD)						
**Initial**	**After 7 days**	**After 14 days**	**After 21 days**	**After 30 days**	**After 8 weeks**	**After 12 weeks**
6.67 ± 0.02	6.66 ± 0.08	6.77 ± 0.02	6.71 ± 0.03	6.73 ± 0.10	6.68 ± 0.03	6.65 ± 0.05
6.70 ± 0.03	6.63 ± 0.02	6.74 ± 0.02	6.69 ± 0.04	6.72 ± 0.02	6.66 ± 0.04	6.63 ± 0.05

Detailed physical stability:

Mechanical Stability

The physical stability of the oleogel was assessed by repeated centrifugation at 3000 rpm for 30 min throughout the storage period in order to evaluate its resistance to accelerated mechanical stress. After each centrifugation cycle, the samples were visually inspected for evidence of phase separation, oil exudation, or structural disruption. No such changes were observed, and the oleogel retained a homogeneous and cohesive appearance throughout the study. These findings indicate that the gel network efficiently retained the lipid phase and exhibited good resistance to mechanical stress, supporting the physical stability of the formulation.

Therml Stability

The thermal stability of the oleogel was evaluated under refrigerated (4 ± 2 °C), ambient (25 ± 2 °C), and accelerated (37 ± 2 °C) storage conditions over the monitoring period (Table S2). At 4 °C, the oleogel maintained its structural integrity, although a slight increase in firmness was observed, which may be associated with increased lipid crystallization at low temperature. Storage at 25 °C did not induce noticeable changes in appearance, consistency, spreadability, color, or odor, indicating excellent stability under normal storage conditions. At 37 °C, a slight reduction in consistency was observed, suggesting partial relaxation of the gel network. Nevertheless, the formulation remained homogeneous, with no evidence of phase separation or oil exudation, while its color and odor remained unchanged. Overall, the oleogel demonstrated good thermal stability across the investigated temperature range.

Freeze–Thaw Stability

The stability of the oleogel was further evaluated through 5 freeze-thaw cycles performed between – 20 °C and 30 °C, with each cycle lasting 24 hours. The results of the freeze-thaw test are summarized in Table S3 from Supplementary Material. This test was designed to simulate temperature fluctuations that may occur during storage.

After 5 successive cycles, the oleogel maintained its structural integrity, with no visible phase separation or oil exudation. The formulation remained homogenous, and no significant changes in color or odor were observed. A slight variation in texture was noted, consisting of a marginal increase in firmness after exposure to lower temperatures, followed by recovery upon returning to ambient conditions. This behavior can be attributed to reversible changes within the gel network.

Table S2. Effect of storage temperature on the physical stability of the oleogel.

**Parameter**	**4 ± 2° C**	**25 ± 2° C**	**37 ± 2° C**
Appearance	Semi-solid, slightly firmer	Semi-solid, uniform	Semi-solid, slightly softened
Color	Stable, no visible changes	Stable, characteristic yellow-orange	Slightly reduced intensity (negligible)
Odor	No changes detected	No changes detected	No rancid odor detected
Homogeneity	Maintained; no phase separation	Maintained; no phase separation	Maintained; no phase separation
Texture	Slightly increased firmness	Smooth and consistent	Slight decrease in firmness
Consistency	More rigid structure	Optimal consistency	Slightly reduced consistency
Film-forming ability	Slightly reduced	Optimal	Slightly increased
Stability assessment	Good stability at low temperature	Excellent stability under normal conditions	Good stability under accelerated conditions

Table S3. Freeze-thaw stability of the oleogel.

**Cycle number**	**Condition (°C)**	**Observations**
1	– 20 → + 30	No phase separation, slight increase in firmness at – 20 °C
2	– 20 → + 30	Homogenous structure maintained; texture returned after warming
3	– 20 → + 30	No visible changes; stable color and odor
4	– 20 → + 30	Slight reversible increase in consistency at low temperature
5	– 20 → + 30	No oil exudation; structure remained intact

Table S4. Rheological tests of the oleogel.

Time	**Viscosity *η* (mPa·s, mean ± SD)**		
	100 rpm	150 rpm	200 rpm
T0	2448 ± 5.6	2405 ± 12.5	2545 ± 5.6
T7	2397 ± 3.0	2387 ± 3.0	2466 ± 4.6
T14	2377 ± 3.6	2357 ± 2.6	2425 ± 5.6
T21	2327 ± 3.6	2296 ± 3.6	2378 ± 2.0
T30	2194 ± 2.0	2158 ± 2.0	2214 ± 4.0
T56	2208 ± 2.0	2164 ± 2.0	2218 ± 2.0
T84	2230 ± 2.0	2185 ± 2.0	2240 ± 2.0

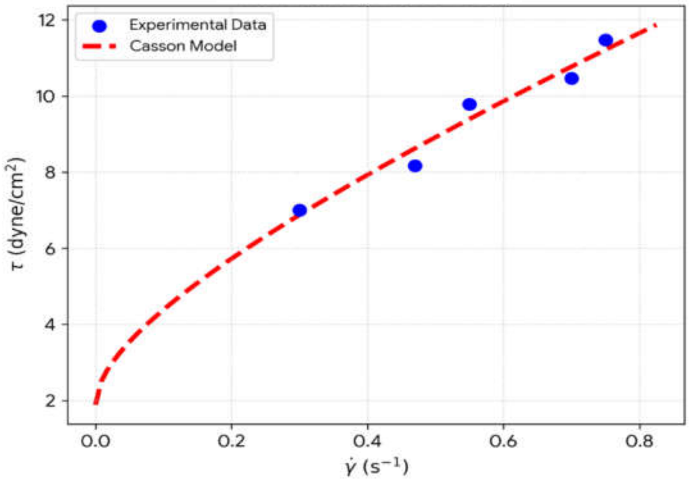

Figure S6. Experimental shear stress versus shear rate data fitted with the Casson rheological model. The high coefficient of determination (R^2 = 0.9795) demonstrates the suitability of the Casson model for describing the flow behavior of the oleogel. The model yielded a Casson yield stress (τ_0) of 0.324 Pa and a Casson plastic viscosity (ηc) of 3.73 cP·s, confirming the presence of a structured network requiring a finite stress to initiate flow.

Table S5. Experimental values used for Casson plot construction (Figure 4), obtained from rotational rheological measurements of the oleogel formulation

Speed (RPM)	Shear Rate ($\gamma^{\cdot}$, s^{-1})	Shear Stress (τ, dyne/cm^2)	$\sqrt{\gamma^{\cdot}}$ ($s^{-1/2}$)	$\sqrt{\tau}$ ($dyne^{1/2}$/cm)
0.1	0.75	11.47	0.8660	3.3867
0.2	0.70	10.46	0.8367	3.3867
0.5	0.55	9.78	0.7416	3.1273
0.8	0.47	8.17	0.6856	2.8583
1	0.30	7.00	0.5477	2.6458

Casson model calculations :the yield stress is determined by squaring the intercept (c) obtained from the linear equation:

$\tau_0=(c)^2= (1.3803)^2=1.9051$ dyne/cm^2

Converting to SI Units (Pa):

Since 1 dyne/cm^2=0.1 Pa:

$\tau_0=1.9051\times0.1=0.1905$ Pa

Casson Plastic Viscosity:

$\eta c=(m)^2= (2.2779)^2=5.189$ cP·s^{-1} =0.5189 Pa·s

Our dataset shows that as the shear rate decreases (from 0.75 down to 0.30 s^{-1}), the apparent viscosity changes from 1530 cP to 401 cP. This confirms a prominent non-Newtonian pseudoplastic flow behavior, highly typical for structural lipid networks.

The calculated yield stress value (τ_0=0.1905 Pa) represents the strength of the three-dimensional crystalline fat network trapping the liquid oil inside the oleogel. Below this stress value, the oleogel behaves as a viscoelastic solid, holding its structure. When an external force exceeding 0.1905 Pa is applied, the crystalline network reversibly or irreversibly breaks down, causing the material to transition into a flowing liquid state.

Table S6. Structural recovery profile of the oleogel (mean ± SD, n = 3).

Time point	Viscosity (mPa·s)
Initial viscosity η_0 (20 rpm, 2 min)	2235.67 ± 34.96
After shear treatment $\eta_{breakdown}$ (200 rpm, 5 min)	1341.33 ± 8.50
Recovery after 1 min (20 rpm)	2374.67 ± 6.81
Recovery after 3 min (20 rpm)	2455 ± 3.00
Recovery after 5 min (20 rpm)	2556.67 ± 9.02
Recovery after 10 min (20 rpm)	2611.33 ± 9.02
Recovery (%)	116.80%

Table S7. Structural recovery parameters of the oleogel determined by the three interval thixotropy test (3 ITT).

Parameter	Formula	Value (%)
Viscosity loss	$Viscosity\ loss\ (\%) = \frac{\eta_0-\eta_{breakdown}}{\eta_0} x100$	40.01
Structural recovery	$Structural\ Recovery\ (\%) = \frac{\eta_{10}}{\eta_0} x100$	116.80
Recovery efficiency	$Recovery\ Efficiency\ (\%) = \frac{\eta_{10}-\eta_{breakdown}}{\eta_0-\eta_{breakdown}} x100$	141.96

Table S8. The results of spreadability for the oleogel.

Nr. crt.	Mass (grams)	Spreadabilty (mm/g) (mean ± SD)	Spreading area (mm^2)
1	10	22.40 ± 0.04	394.08
2	20	23.44 ± 0.06	431.57
3	30	23.74 ± 0.05	442.67
4	50	25.94 ± 0.07	528.34
5	100	28.88 ± 0.03	654.89
6	150	32.05 ± 0.09	806.86
7	200	33.44 ± 0.06	878.40
8	250	35.58 ± 0.04	983.15

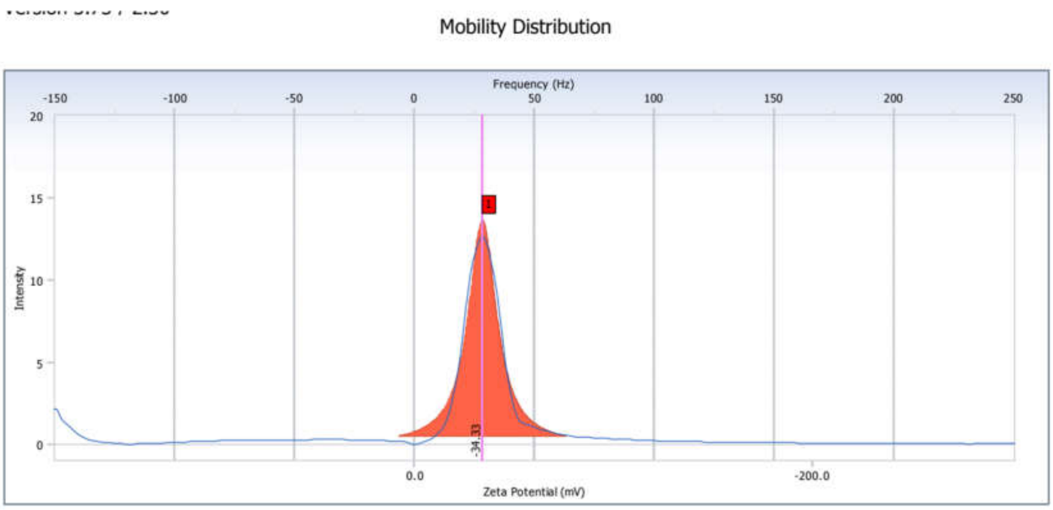

Measurement Results

Zeta Potential	: -34.33	(mV)	Doppler shift	: 28.52	(Hz)
Mobility	: -2.676e-004	(cm²/Vs)	Base Frequency	: 125.6	(Hz)
Conductivity	: 0.0053	(mS/cm)	Conversion Equation	: Smoluchowski	

Figure S7. Zeta potential distribution of the oleogel dispersed in distilled water. The predominance of negatively charged particles indicates the presence of ionizable surface groups and supports the colloidal stability of the aqueous dispersion.

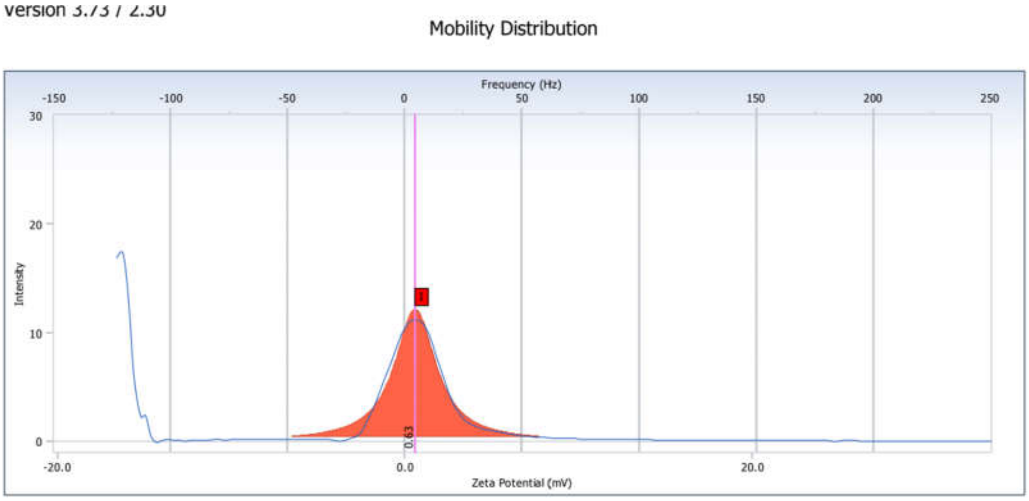

Measurement Results

Zeta Potential	: 0.63	(mV)	Doppler shift	: 4.65	(Hz)
Mobility	: 4.890e-006	(cm²/Vs)	Base Frequency	: 121.8	(Hz)
Conductivity	: 0.0003	(mS/cm)	Conversion Equation	: Smoluchowski	

Figure S8. Zeta potential distribution of the oleogel dispersed in ethanol. The measured zeta potential was +0.63 mV, indicating a near-neutral surface charge and limited electrostatic stabilization. The narrow distribution suggests a relatively homogeneous

dispersed population, while the colloidal stability is likely maintained predominantly through steric and intermolecular interactions within the oleogel matrix.

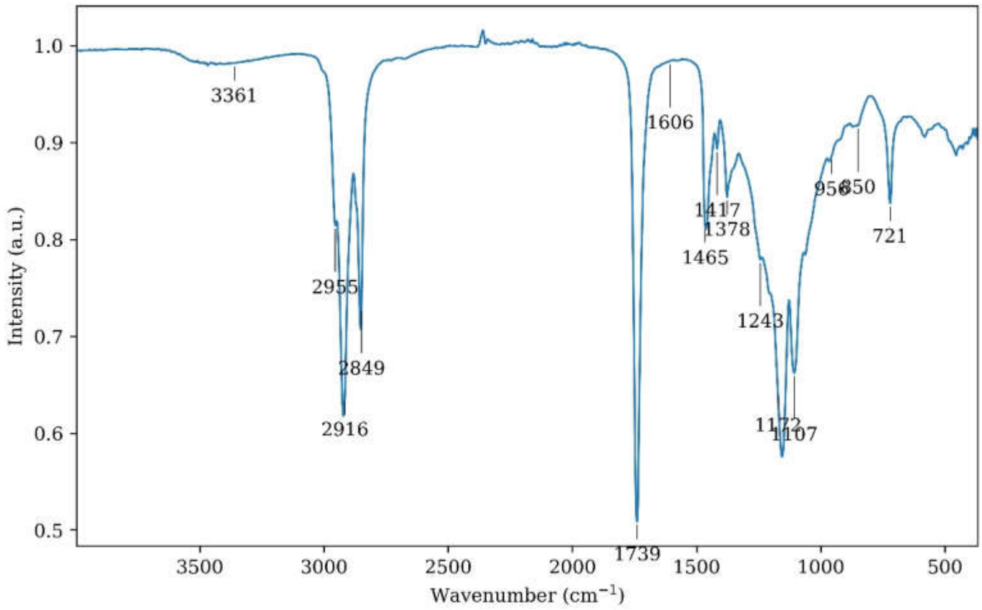

Figure S9. ATR–FTIR spectrum of the oleogel deposited on a silicon substrate.

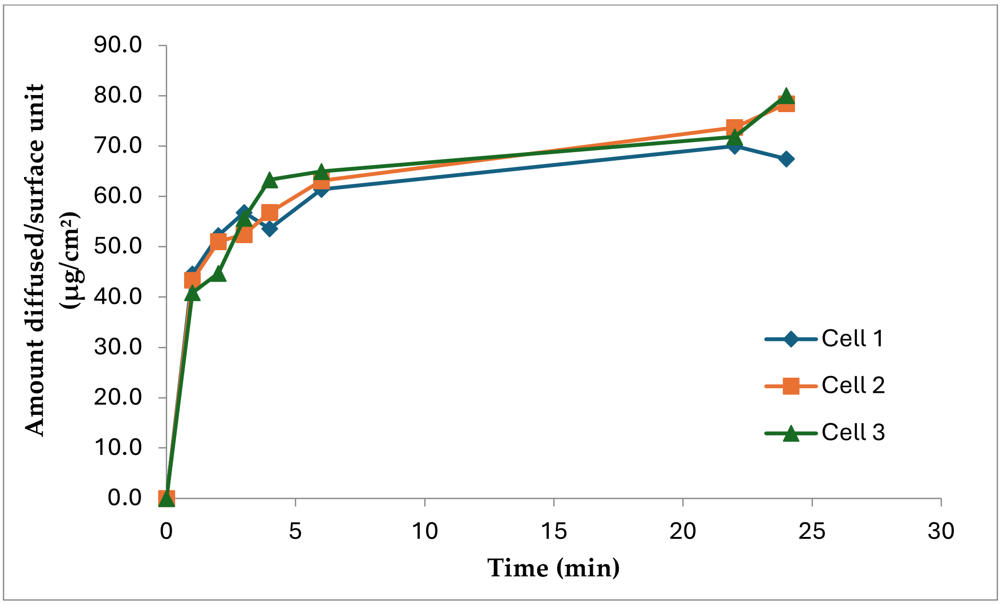

Figure S10. Individual release profiles of total phenolic compounds obtained from three independent Franz diffusion cells.

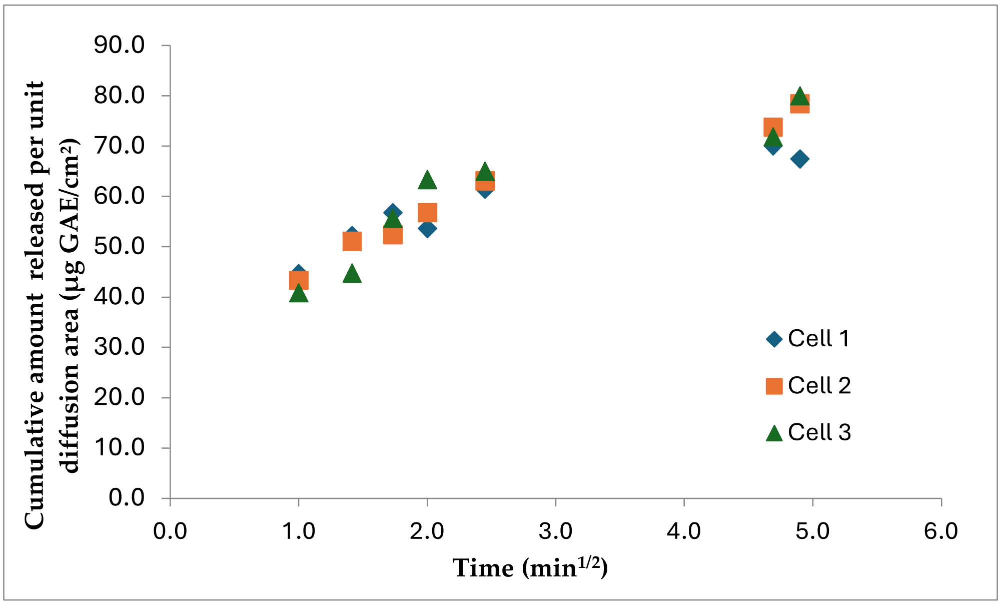

Figure S11. Higuchi plot showing the relationship between the cumulative amount of phenolic compounds released per unit diffusion area (μg GAE/cm²) and the square root of time.

Table S9. Thermal stress conditions of oleogel.

Condition	Time of test	Purpose
4 ± 2 °C (refrigerator)	T0, T30, T56, T84	Evaluation of physical stability under low-temperature conditions; assessment of potential crystallization or phase changes in the oleogel structure
25 ± 2 °C (room temperature)	T0, T30, T56, T84	Assessment of formulation stability under normal storage conditions; monitoring of physicochemical and organoleptic properties over time
37 ± 2 °C (incubator)	T0, T30, T56, T84	Evaluation of stability under accelerated condions; investigation of oxidative processes, structural changes, and potential degradation of bioactive compounds
